# The E3 ligase HECTD4 regulates COX-2-dependent tumor progression and metastasis

**DOI:** 10.1073/pnas.2425621122

**Published:** 2025-08-06

**Authors:** Joanna A. Vuille, Cem Tanriover, Ezgi Antmen, Douglas S. Micalizzi, Richard Y. Ebright, Sambhavi Animesh, Robert Morris, Soroush Hajizadeh, Zachary J. Nicholson, Hunter C. Russell, Eric F. Zaniewski, Ben S. Wittner, Ben K. Wesley, Ji Eun Kwak, Julian Grünewald, Regan N. Szalay, Douglas B. Fox, Min Yang, J. Keith Joung, Doga C. Gulhan, Andrew E. H. Elia, Wilhelm Haas, Eugene Oh, Shyamala Maheswaran, Daniel A. Haber

**Affiliations:** ^a^Krantz Family Center for Cancer Research, Massachusetts General Hospital Cancer Center and Harvard Medical School, Charlestown, MA 02129; ^b^Department of Oncology, Centre Hospitalier Universitaire Vaudois, Lausanne University, Lausanne 1005, Switzerland; ^c^Center for Computational and Integrative Biology, Massachusetts General Hospital, Charlestown, MA 02129; ^d^Department of Pathology, Massachusetts General Hospital and Harvard Medical School, Boston, MA 02115; ^e^HHMI, Chevy Chase, MD 20815; ^f^Arena BioWorks, Cambridge, MA 02142; ^g^Department of Radiation Oncology, Massachusetts General Hospital and Harvard Medical School, Boston, MA 02114

**Keywords:** breast cancer, metastasis suppressor genes, tumor suppressor gene, ubiquitination, anchorage independence

## Abstract

E3 ubiquitin ligases mediating turnover of proteins engaged in cancer progression point to key regulatory nodes. To uncover modifiers of metastatic competency, we conducted an in vivo genome-wide CRISPR-inactivation screen using cultured breast circulating tumor cells, following intravascular seeding and lung colonization. We identified HECTD4, a previously uncharacterized gene encoding a conserved potential homologous to E6AP C-terminus domain–containing ubiquitin transferase, as a potent tumor and metastasis suppressor. We show that purified HECTD4 mediates ubiquitin conjugation in vitro, and proteomic studies combined with ubiquitin remnant profiling identify a major degradation target as the prostaglandin synthetic enzyme cyclooxygenase-2 (*COX-2*; *PTGS2*). In addition to COX-2 itself, HECTD4 targets its regulatory kinase MKK7. In breast cancer models, HECTD4 expression is induced as cells lose adherence to the matrix, and its depletion massively increases COX-2 expression, enhancing anchorage-independent proliferation and tumorigenesis. Genetic or pharmacologic suppression of COX-2 reverses the protumorigenic and prometastatic phenotype of HECTD4-depleted cells. Thus, HECTD4 encodes an E3 ubiquitin ligase that downregulates COX-2 suppressing anchorage independence in epithelial cancer cells.

## Significance

A genome-wide CRISPR-inactivation screen identified the previously uncharacterized E3 ubiquitin ligase HECTD4, as a tumor and metastasis suppressor, with COX-2 as its major degradation target. The protumorigenic and prometastatic effect of HECTD4 suppression depends on COX-2 stabilization, which is critical for anchorage-independent growth, providing a basis for investigating COX-2 inhibition to prevent metastatic recurrence.

Circulating tumor cells (CTCs) are shed from cancerous lesions into the bloodstream and constitute precursors of metastasis. These circulating cancer cells exhibit the heterogeneity acquired by advanced tumors, as they initially respond and then progress following therapeutic interventions by adapting to loss of matrix adhesion, high oxygen tensions, and circulatory stress in the bloodstream (1). By using a microfluidic platform to deplete leukocytes from blood samples and preserve viable CTCs, we have successfully established long-term cultures of breast cancer CTCs, maintained under anchorage-independent, hypoxic, stem cell culture conditions (2, 3). While cultured CTCs produce orthotopic mammary gland tumors in immunosuppressed mice, they remarkably fail to initiate lung tumors following direct tail vein intravenous inoculation (4). Identifying factors that modulate the functional properties of cultured CTCs may, thus, identify strategies to suppress blood-borne cancer metastasis.

Inflammatory mediators secreted into the microenvironment may mediate both autocrine and microenvironmental effects on tumorigenesis. The cyclooxygenase COX-2, itself triggered by inflammatory stimuli, catalyzes the synthesis of prostaglandins such as PGE2, contributing to cancer cell proliferation, apoptosis resistance, angiogenesis, and fibrosis (5, 6). Consistent with an important role in tumor initiation, seminal epidemiological studies of the COX-2 inhibitor Celecoxib demonstrated striking benefits in colorectal cancer prevention, but at the risk of increased thrombosis and cardiovascular events, leading to the discontinuation of cancer prevention studies (7, 8). A role for COX-2 suppression of metastasis through nonsteroidal anti-inflammatory drugs (NSAID) has also been suggested in epidemiological studies (9), but it is unclear whether this reflects tumor cell–specific effects, anti-inflammatory activity, or alterations in platelet aggregation. Recent and ongoing clinical studies have focused on aspirin use to suppress metastatic recurrence in patients with a history of cancer, with ongoing consideration of predictive biomarkers, patient stratification, drug dosage, and duration of follow-up (9–11). The potential utility of COX-2 inhibitors in the suppression of metastasis thus remains to be resolved.

Here, we conducted a genome-wide in vivo CRISPR-inactivation (CRISPR-i) screen using cultured breast CTCs, identifying *HECTD4,* a previously uncharacterized member of the Homologous to E6AP C-Terminus (HECT) ubiquitin ligase gene family, as a suppressor of tumor initiation in a model of lung metastasis. We show that HECTD4 specifically promotes the ubiquitination and degradation of COX-2, an effect that is most dramatic as breast cancer cells lose cell adhesion. *COX-2* knockdown within breast cancer cells abrogates the anchorage-independent survival benefit of *HECTD4* suppression, consistent with a tumor cell-intrinsic role for COX-2 activity in metastasis initiation.

## Results

### Suppression of CTC-Mediated Metastasis by HECTD4 in a CRISPR-i Screen.

**Fig. 1.**
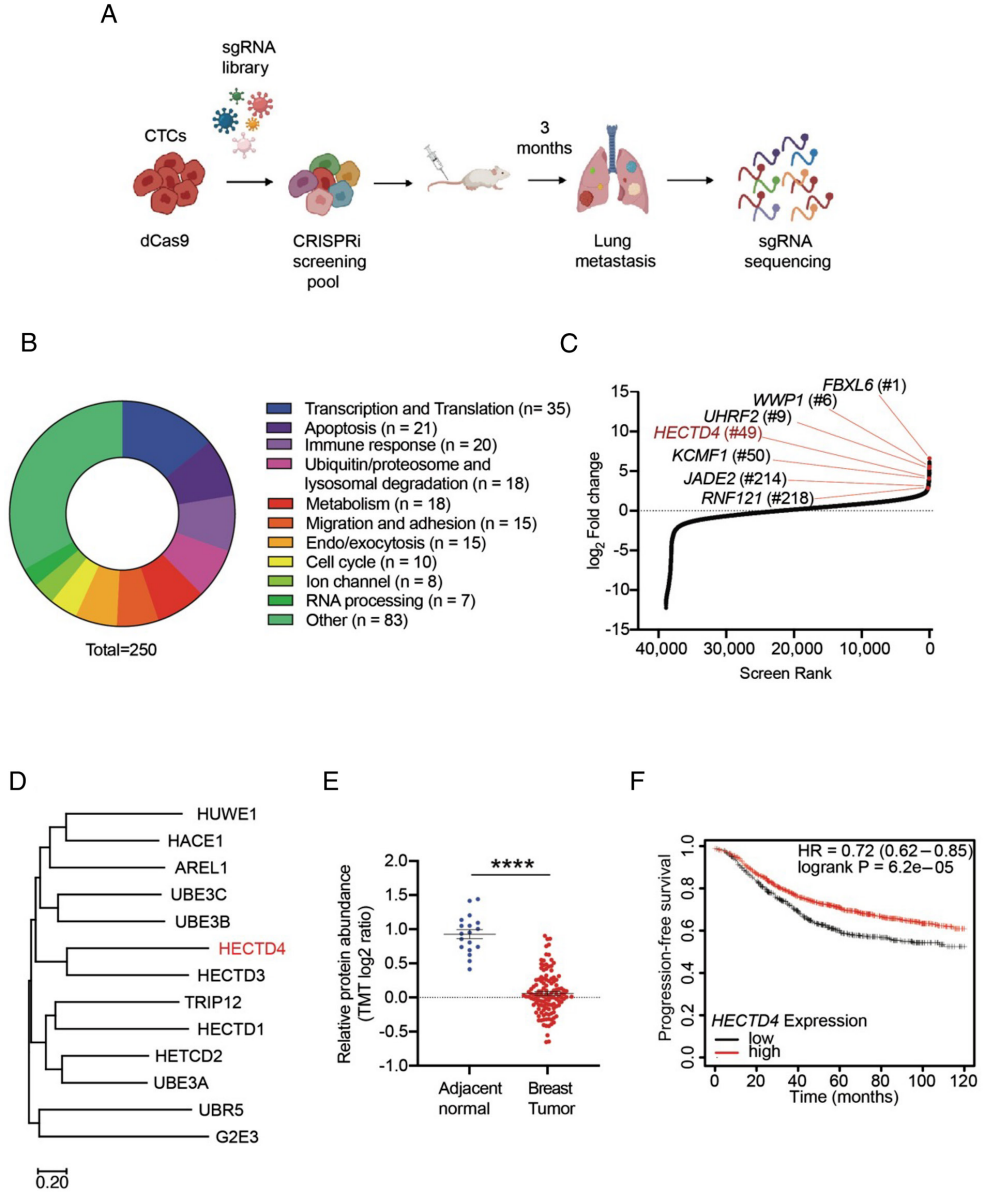
Suppression of CTC-mediated metastasis by HECTD4 in a CRISPR-i screen. (*A*) Schematic diagram of the in vivo CRISPR inactivation screen in CTCs. (*B*) Classification of known functions of the top 250 genes identified in the in vivo CRISPRi screen. (*C*) Distribution of ranked screen scores, according to the fold change compared to the input (log2 FC). The top genes classified as E3 ubiquitin ligases are indicated with *HECTD4* highlighted in red. (*D*) The evolutionary tree showing the 13 members of the HECT subfamily. The distances are in the units of the number of amino acid substitutions per site. (*E*) Proteomics data from breast tumor tissues showing a reduction in HECTD4 protein in tumors compared with normal breast tissue. Significance was calculated using the unpaired Student *t* test. (*F*) Kaplan–Meier plot shows that breast cancer patients with high *HECTD4* expression (n = 1,433) in their tumors have improved progression-free survival compared with those with low *HECTD4* expression. Significance determined by log-rank (Mantel–Cox) test. (*P < 0.05, **P < 0.01, ***P < 0.001, and ****P < 0.0001).

To identify negative regulators of CTC-mediated metastasis, we introduced a genome-wide CRISPR-i library into BRx-142 cells, a CTC-derived cell line generated from a blood sample of a patient with advanced, refractory hormone receptor-positive (HR+) breast cancer (3). BRx-142 CTCs are highly tumorigenic following orthotopic inoculation in NSG immunosuppressed mice, yet they fail to produce sustained metastases in the lungs following intravenous tail vein inoculation (4). The cultured CTCs were first transduced to stably express a catalytically inactive Cas9 (dCas9) fused to a transcription repressor domain (KRAB), followed by transduction with a sgRNA library targeting >18,000 genes, with 3 guides per gene at an MOI of 0.3, to achieve a representation of 300 to 350 cells per sgRNA (12). NSG immunodeficient mice (n = 15) were inoculated by tail vein with the engineered Brx142 CTCs, and their lungs were harvested after 3 mo, followed by bulk sequencing to score sgRNA enrichment, compared with sample input (Fig. 1*A*). To mitigate the heterogeneity of guide representation, we pooled sequences from all mice, excluded low-input sgRNAs with fewer than 100 absolute reads in the viral pool (148 sgRNAs), and rank-ordered genes based on their most highly enriched gRNA (*Materials and Methods*). Among the high-scoring genes were known mediators of tumor progression, including genes involved in transcription/translation (n = 35) and apoptosis (n = 21) (Fig. 1*B*). Remarkably, multiple hits identified genes implicated in proteolytic degradation, with seven E3 ubiquitin ligases ranking among the top 250 hits. Six of these genes encode the E3 ligases *FBXL6, WWP1, UHRF2, KCMF1, JADE2, and RNF121,* each with previously well-defined substrates and a seventh (ranked 49), *HECTD4*, whose substrate and function is unknown (Fig. 1*C*).

Among the three major gene families of E3 ubiquitin ligases, the HECT family encompasses 13 proteins sharing a HECT domain, which catalyzes the transfer of the ubiquitin moiety onto target proteins (13). HECTD4 shares a catalytic HECT domain with 12 other members of this family of E3 ubiquitin ligases (Fig. 1*D*). HECT family members have known targets implicated in breast cancer development and progression (14), but HECTD4 has remained uncharacterized, with no known function or other recognizable functional domain and noteworthy for its very large size (460 kD). Inherited genetic variants of *HECTD4* are associated with neurological disease and increased risk for diabetes and hypertension (13).

To support a potential role in cancer, we first explored clinical datasets, observing that both *HECTD4* mRNA and protein expression are considerably reduced in primary and metastatic breast cancers, compared with normal breast tissue (Fig. 1*E* and *SI Appendix*, Fig. S1*A*) (15). Moreover, lower *HECTD4* expression in breast cancer is associated with reduced progression-free survival (Fig. 1*F*) (16). Among histological subsets of breast cancer, *HECTD4* expression is lowest in the most aggressive subtype, triple-negative breast cancer (TNBC), a proportion of which display allelic losses around the gene locus (*SI Appendix*, Fig. S1 *B* and *C*). In addition to breast cancer, *HECTD4* expression is reduced in multiple other tumor types compared with corresponding normal tissues, including glioblastoma and esophageal cancers (*SI Appendix*, Fig. S1 *D* and *E*). We therefore selected *HECTD4* as the CRISPR-i hit for detailed analysis.

### Anchorage-Independent Proliferation and Increased Tumorigenesis following *HECTD4* Depletion.

**Fig. 2.**
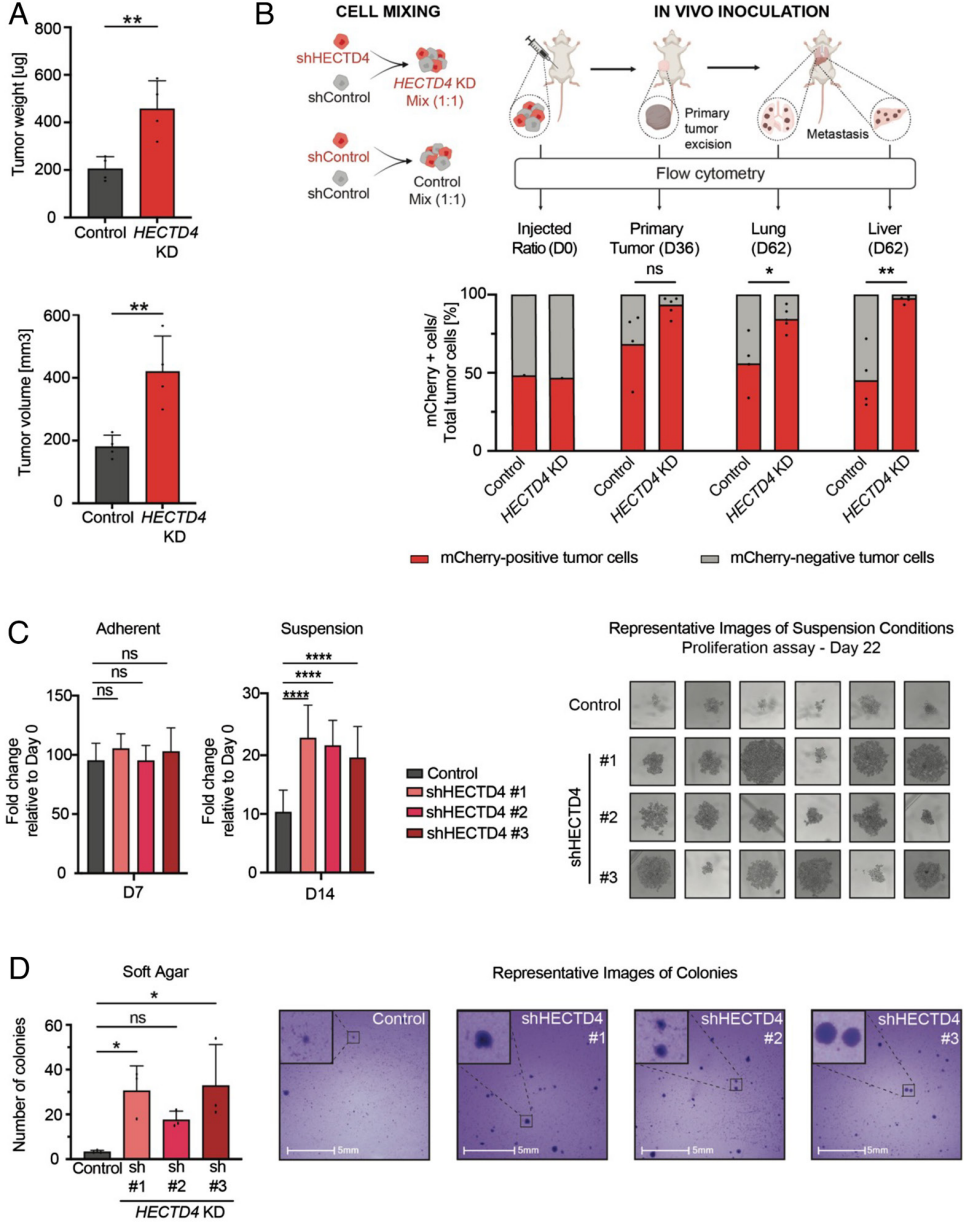
Anchorage-independent proliferation and increased tumorigenesis following *HECTD4* depletion. (*A*) The initial in vivo validation experiment comparing *HECTD4*-KD (sh #1) cells with scrambled control cells. MDA-MB-231 cells with *HECTD4*-KD (sh #1) or scrambled control were injected separately into the mammary fat pad of NSG mice (n = 4 mice for the control group, n = 4 mice in *HECTD4*-KD group). Primary tumors were harvested after 34 d and assessed for tumor weight and tumor volume. The bar graph shows tumor weights (*Upper*) and volumes (*Lower*) measured on day 34. Error bars represent mean ± SD. Significance was calculated using the unpaired Student *t* test. The mRNA levels of *HECTD4* in the cells used for this experiment are shown in *SI Appendix*, Fig. S2*A*. (*B*) Schematic representation of the tumor cells mixing experiment to interrogate the effects of HECTD4 on tumorigenesis. shControl cells (GFP-shControl; *HECTD4*-WT, shown in gray) were mixed with either GFP-tagged and mCherry labeled *HECTD4*-knockdown cells [GFP-mCherry-*HECTD4*-KD, shown in red] (experimental cohort) or with GFP-tagged and mCherry labeled shControl cells [GFP-mCherry-shControl; *HECTD4*-WT, shown in red] (control cohort). A 1:1 mixture of GFP-shControl and GFP-mCherry-*HECTD4*-KD or GFP-shControl and GFP-mCherry-shControl was inoculated into the mammary fat pad of immunodeficient NSG mice (n = 4 mice for control mixture; n = 5 mice for KD mixture, see *Materials and Methods* for more detail). The mixtures were analyzed by flow cytometry right before inoculation to ensure that each of the populations was equally represented in the mixture inoculated into the mammary fat pad on day 0. The primary tumors were resected after 36 d via survival surgery of the mice and the colonized organs—the lungs and livers—were harvested after 62 d. The ratio of the GFP: mCherry populations was analyzed by flow cytometry in the control and experimental cohorts (*Upper*). The bars represent the percentage of green (GFP-shControl, shown in gray) and red (GFP-mCherry-shControl or GFP-mCherry-*HECTD4*-KD, shown in red) cells in the primary and metastatic tumors. Dots represent the ratio of m-Cherry-tagged (manipulated) cells compared with m-Cherry-negative (control) cells within each tumor. Significance was calculated with unpaired the Student *t* test (*Lower*). The mRNA levels of *HECTD4* in the cells used for this experiment are shown in *SI Appendix*, Fig. S2*B*. (*C*) In vitro growth of *HECTD4*-depleted MDA-MB-231 cells compared to scrambled control cells under adherent 2D-culture on day 7. Error bars represent mean ± SD. Significance was calculated using two-way ANOVA, Tukey’s multiple comparison test (day 7) (*Left*). In vitro growth of *HECTD4*-depleted MDA-MB-231 cells compared to scrambled control cells under anchorage-independent suspension conditions (ultralow-adherent plates) on day 14. Error bars represent mean ± SD. Significance was calculated using the two-way ANOVA, Tukey’s multiple comparison test (day 14) (*Middle*). Representative photomicrographs of MDA-MB-231 cells grown in anchorage-independent suspension conditions (ultralow-adherent plates) on day 22 (*Right*). The mRNA levels of *HECTD4* in the cells used for this experiment are shown in *SI Appendix*, Fig. S2*A*. (*D*) Single MDA-MB-231 cells with either *HECTD4* depletion or scrambled control were seeded on soft-agar coated 6-well plates (10,000 cells/well). After 2 wk, the colonies were stained with crystal violet, imaged, and the number of colonies was counted using the HALO software. Error bars represent mean ± SD. Significance was calculated using the one-way ANOVA test, with Dunnett’s multiple comparison test (*Left*). Representative images of these colonies (*Right*). The mRNA levels of *HECTD4* in the cells used for this experiment are shown in *SI Appendix*, Fig. S2*F*. (*P < 0.05, **P < 0.01, ***P < 0.001, and ****P < 0.0001).

To validate the effect of *HECTD4* suppression, we turned to the TNBC cell line MDA-MB-231, which is highly invasive and metastatic, as well as genetically manipulable. Despite their high baseline tumorigenesis, MDA-MB-231 cells with shRNA-mediated *HECTD4* knockdown (>80%) doubled in tumor size in immunosuppressed NSG mice, compared with sh-scramble controls (457.1 µg ± 118.1 vs. 204.9 µg ± 51.01, *P* = 0.0078; Fig. 2*A* and *SI Appendix*, Fig. S2*A*). To further quantify this effect, we performed chimeric tumorigenicity experiments by mixing GFP- and mCherry-tagged tumor cells (Fig. 2 *B*, *Upper* and *SI Appendix*, Fig. S2*B*). In a control experiment, a 1:1 mixture of GFP- and mCherry-tagged MDA-MB-231 cells (GFP-shControl; *HECTD4*-WT vs. GFP-mCherry-shControl; *HECTD4*-WT) (n = 4-5 per group) shows persistence of the 1:1 ratio in primary tumor (orthotopic mammary fat pad), comparable with the ratio on day 0 input cell population, as measured by FACS analysis. Following resection of the primary tumor (day 36 postinoculation) to allow for the development of metastases (day 62), the mCherry/GFP ratio remains constant in lung and liver metastases. In marked contrast, inoculation of a 1:1 mixture of control and *HECTD4*-KD cells (GFP-shControl; *HECTD4*-WT vs. GFP-mCherry-*HECTD4*-KD; *HECTD4*-KD) shows a dramatically increased representation of the *HECTD4*-deficient cells in both primary and metastatic lesions. In all primary tumors, *HECTD4*-KD cells constituted >80% of cells, and as high as >95% in three of five tumors. Similarly, *HECTD4*-KD cells contributed to >70% of metastatic tumor cells in the lungs and >90% in the liver (Fig. 2 *B*, *Lower* and *SI Appendix*, Fig. S2*D*). Thus, even in the highly invasive MDA-MB-231 breast cancer cells, suppression of *HECTD4* further enhances both primary and metastatic tumorigenesis.

Despite the striking effect of *HECTD4* knockdown in vivo, it has no effect on in vitro proliferation of MDA-MB-231 cells under standard adherent 2D culture conditions (Fig. 2 *C*, *Left* and *SI Appendix*, Fig. S2*A*). Remarkably, however, *HECTD4*-KD dramatically promotes anchorage-independent growth of these cells upon plating onto low-adherence plates, where they form large spheroid-like structures, that are not present in *HECTD4*-expressing control cells (Fig. 2 *C*, *Middle*, *Right*). *HECTD4*-KD cells grown in suspension show increased PCNA expression, consistent with their increased proliferation (*SI Appendix*, Fig. S2*E*). The increased anchorage-independent proliferation in suspension by *HECTD4*-KD cells is also evident by increased colony formation in soft agar assays, a commonly used hallmark of tumorigenesis (Fig. 2 *D*, *Left*, *Right* and *SI Appendix*, Fig. S2*F*).

To assess the effect of *HECTD4* knockdown in an additional breast cancer cell line, we transduced MDA-MB-468 cells with either sh-scramble controls or shHECTD4 along with a luciferase reporter plasmid (Luciferase+ MDA-MB-468) (*SI Appendix*, Fig. S3*A*). Consistent with our findings in MDA-MB-231 cells, *HECTD4*-deficient MDA-MB-468 cells also demonstrated increased anchorage-independent growth under low-adherence suspension conditions (*SI Appendix*, Fig. S3*B*). In addition, tail vein injections of these cells into NSG immunosuppressed mice resulted in the formation of larger lung lesions in the *HECTD4*-KD group compared to controls, suggesting improved metastatic colonization of the lungs in the absence of HECTD4 (*SI Appendix*, Fig. S3*D*). Taken together, these findings show that depletion of *HECTD4* results in enhanced anchorage-independent proliferation in vitro as well as increased tumorigenesis and metastasis in vivo.

### Regulation of COX-2 by HECTD4.

**Fig. 3.**
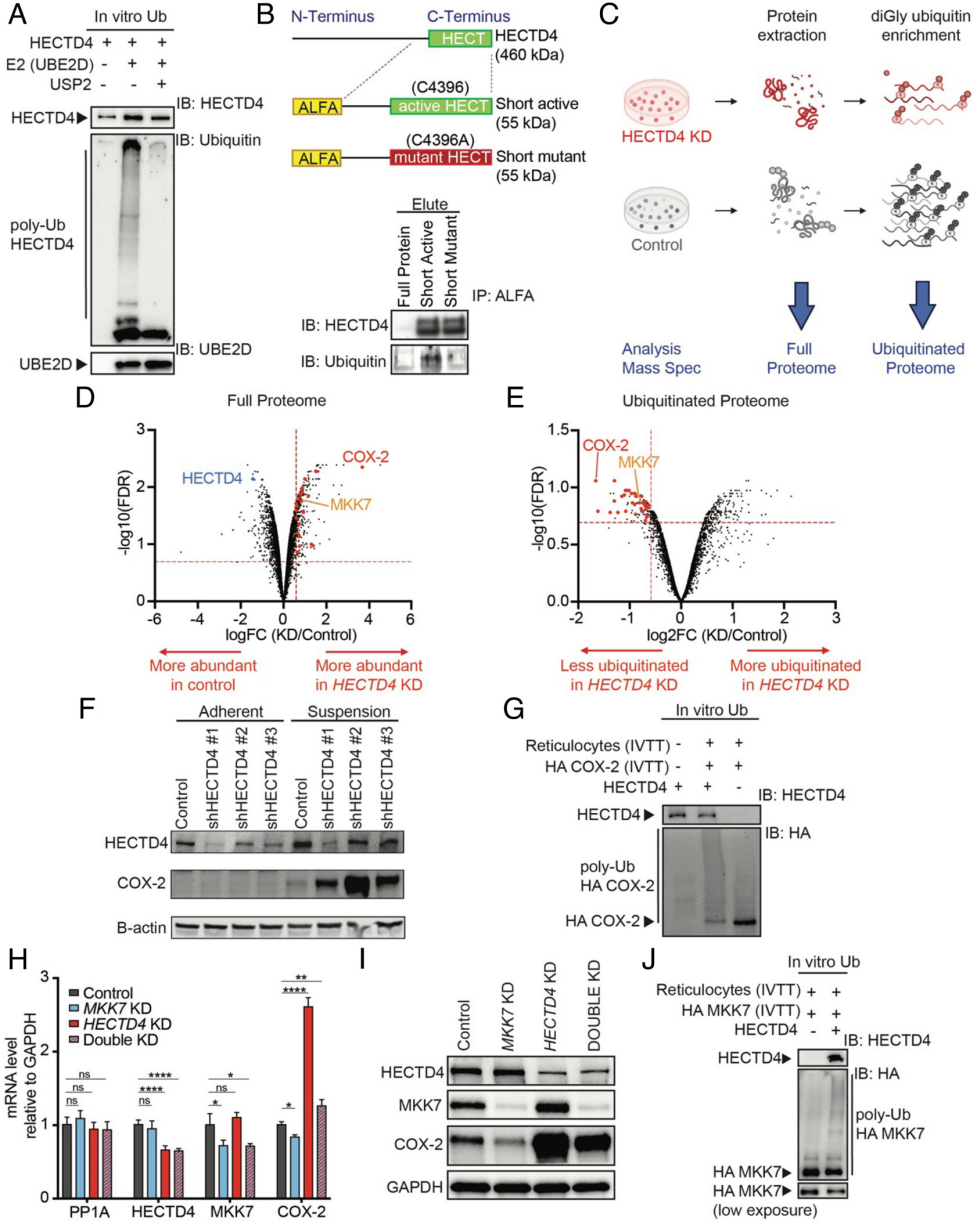
HECTD4 modulates the stability of COX-2 and its regulatory kinase MKK7. (*A*) HECTD4 catalyzes ubiquitin chain formation in vitro. Active HECTD4 was immunoprecipitated from freshly lysed cells expressing the full-length alfa-tagged HECTD4 using a nanobody against the alfa-tag. Addition of E1, UBE2D, ATP, and ubiquitin results in the formation of polyubiquitin conjugates. The addition of the deubiquitinating enzyme USP2 leads to the loss of the polyubiquitin chain. The potential reaction products were detected by western blotting against HECTD4, ubiquitin, and UBE2D. (*B*) Schematic representation of the cloning strategy of the C-terminal end of HECTD4. The short active product contains the reference sequence of HECT and 380 nucleotides upstream, including the putative catalytic cysteine, C4396, in green. In the short mutant form, this cysteine was mutated into an alanine (C4396A). To facilitate the immunoprecipitation and increase its specificity, both products were tagged at the N terminus with an ALFA tag. The resultant protein has an expected molecular weight of 55 kDa (*Upper*). Lysates from cells transfected with the short-active and short-mutant forms of alfa-HECTD4 were flowed through an ALFA-tag activated column. The input lysate, flow-through, and the eluate were blotted and probed with antibodies against HECTD4 and ubiquitin. Untransfected parental cells are shown as control. The elute is shown here; the input lysate and flow-through are shown in *SI Appendix*, Fig. S4*D*. Ubiquitin blotting detects signal from the short active form of HECTD4 but not from the short mutant in which C4396 is converted to Alanine (*Lower*). (*C*) Schematic representation of the quantitative proteomics experiment to identify HECTD4 substrates. *HECTD4*-KD and control cells were cultured under anchorage-independent conditions for 4 d. Cells were lysed in a urea-based buffer, followed by protease digestion of the proteins. A fraction of the complete lysate was conserved for analysis by full proteome mapping. The remaining peptides were purified by reversed-phase, solid-phase extraction. Using an antibody targeting the diGly ubiquitin remnant motif (K-ε-GG), ubiquitinated peptides were then captured by immunoprecipitation and eluted to concentrate them for LC-MS/MS analysis. (*D*) Volcano plot of the complete proteome of *HECTD4*-KD MDA-MB-231 cells versus control cells. The x-axis shows the log2 FC of each identified protein (6266), and the y-axis shows the corresponding –log10 *P* adjusted value. The HECTD4 protein is marked in blue; enriched proteins in the *HECTD4*-KD cells are on the right side of the graph with a positive log2FC. 25 proteins highlighted in red were found to accumulate in the *HECTD4*-KD cells (FC > 1.5 and FDR < 0.25, thresholds in dotted lines) and to have their ubiquitinated forms less abundant in the same cells (FC < −1.5 and FDR < 0.25, thresholds in dotted lines) in the diGly screen. COX-2 ranks as the second most enriched protein (>12-fold increase). (*E*) Volcano plot of the ubiquitinated peptides captured by immunoprecipitation from *HECTD4*-KD cells versus control cells. The x-axis shows the log2 FC of each identified peptide (3311) aligning to 1,453 proteins, and the y-axis shows the corresponding –log10 *P* adjusted value. HECTD4 substrates are expected to be less ubiquitinated in the *HECTD4*-KD cells, thus exhibiting peptides on the left side of the graph with a negative log2FC. The 25 proteins described in Fig. 3*D*. are also highlighted in red. COX-2 is the protein with the most reduced ubiquitination (>threefold reduction compared to normal cells). (*F*) Western blot of MDA-MB-231 cultured in regular adherent conditions (adherent) and placed in suspension (suspension) showing the weak expression of COX-2 in adherent cells compared to suspension cells. *HECTD4*-KD cells express high levels of COX-2 compared with control. HECTD4 protein expression is also upregulated in suspension. (*G*) HECTD4 ubiquitinates COX-2 in vitro. Potential reaction products were detected by western blotting against HECTD4 and HA-tag. The smear demonstrates the presence of a polyubiquitinated HA-COX-2. (*H*) qRT-PCR of *PP1A, HECTD4, MKK7*, and *COX-2* mRNA upon depletion of *HECTD4* only (mix of short hairpins targeting HECTD4), of *MKK7* only (mix of 4 siRNA targeting MKK7, shown in blue), and of a combined KD (mix of shHECTD4 and mix of siMKK7, shown in red and blue stripes). mRNA collected 3 d after siRNA transfection from cells growing in suspension. *COX-2* mRNA levels increase upon *HECTD4* depletion, and *MKK7*-KD reverses it. Error bars represent mean ± SD. Significance was calculated using the one-way ANOVA test, with Dunnett’s multiple comparison test. (*I*) Western blot of a similar experiment described in Fig. 3*H*, with protein lysate harvested after 4 d. (*J*) HECTD4 also ubiquitinates MKK7 in vitro. Potential reaction products were detected by western blotting against HECTD4 and HA-tag. The smear demonstrates the presence of a polyubiquitinated HA-MKK7. Lower exposure of the same gel shown below. (*P < 0.05, **P < 0.01, ***P < 0.001, and ****P < 0.0001).

To first demonstrate that HECTD4 has ubiquitin ligase activity, we used CRISPR prime-editing to engineer an ALFA tag onto the N terminus of endogenous HECTD4, enabling efficient pull-down of native protein using a tag-specific nanobody (*SI Appendix*, Fig. S4*B*). We then performed an autoubiquitination assay in vitro by adding essential components of the ubiquitin cascade, including E1, E2, ubiquitin, and ATP to the purified HECTD4. Western blot analysis of the products shows that the addition of HECTD4 results in the formation of polyubiquitin conjugates, which is characterized by the presence of higher-order molecular weight species of additional ubiquitin moieties. Addition of the deubiquitinating enzyme Ubiquitin Specific Peptidase 2 (USP2) results in the loss of this ubiquitin smear (Fig. 3*A* and *SI Appendix*, Fig. S4*C*). The HECT domain of HECTD4 contains a highly conserved cysteine at its C-terminal end, which, in other family members, is required for ubiquitin-binding and transfer from E2 to the target protein. Accordingly, we cloned a “short-active” form of HECTD4, encoding the ALFA-tagged isolated HECT domain, and mutated the active site cysteine C4396 to alanine to generate a “short-mutant” construct (Fig. 3 *B*, *Upper*). Transfection of the short-active, but not the short-mutant construct, into MDA-MB-231 cells shows autoubiquitination, following immunoprecipitation from cellular extracts (Fig. 3 *B*, *Lower* and *SI Appendix*, Fig. S4*D*).

To identify proteins targeted for degradation by HECTD4, we conducted two complementary proteomic screens, comparing *HECTD4*-KD cells versus controls. In both screens, MDA-MB-231 cells were cultured under low-adherence conditions (Fig. 3*C*). First, we performed quantitative mass spectrometry-based proteomics (17) on total cellular lysate followed by tryptic digest to identify proteins with increased concentration following *HECTD4* knockdown (Fig. 3*D*). Second, we enriched for tryptic peptides containing lysine residues that carry a diGly remnant of ubiquitin (18) at lysine residues and we used mass spectrometry to identify proteins with decreased ubiquitination upon *HECTD4* knockdown (Fig. 3*E*). Looking at the intersection of these two orthogonal assays, a total of 25 proteins satisfied our protein concentration change filter criteria (FC (KD/Control) >2 at an FDR < 0.25). Of these proteins, the cyclooxygenase protein COX-2 was >12-fold more abundant and showed the most pronounced decrease of ubiquitination (>threefold) upon *HECTD4* knockdown (Fig. 3 *D* and *E*).

Western blotting confirms a dramatic increase in COX-2 protein levels in *HECTD4*-KD cells compared to control in MDA-MB-231 cells (*SI Appendix*, Fig. S4 *E* and *F*). Remarkably, this effect is only evident when MDA-MB-231 cells are maintained under anchorage-independent conditions (Fig. 3*F*). COX-2 levels in MDA-MB-231 cells are undetectable under standard 2D culture conditions, but COX-2 expression becomes detectable when cells are placed in suspension; *HECTD4*-KD mediates a significant increase in COX-2 levels under suspension, but not in 2D culture conditions (Fig. 3*F*). Endogenous HECTD4 levels also increase upon anchorage-independent growth, raising the possibility that it may play a physiological role in attenuating the increased COX-2 expression under stressful conditions. The dramatic rise in COX-2 protein levels following depletion of *HECTD4* under anchorage-independent culture is also seen in MDA-MB-468 cells (*SI Appendix*, Fig. S4*G*) as well as two other breast cancer cell lines BT-549 and HCC1143 (*SI Appendix*, Fig. S4 *H* and *I*). To further validate the inverse correlation between HECTD4 and COX-2 proteins, we analyzed a previously published proteomic dataset from 21 different human breast cancer cell lines (19), which confirmed the negative correlation between HECTD4 and COX-2 protein levels (N = 21, r = −0.426, *P* = 0.0541; *SI Appendix*, Fig. S4*J*). Finally, to confirm the direct ubiquitination of COX-2 by native HECTD4, we purified ALFA-tagged endogenous HECTD4 from cells in suspension culture and found that purified HECTD4 can ubiquitinate recombinant HA-tagged COX-2 using in vitro ubiquitination assays. Western blotting demonstrates a smear of polyubiquitinated HA-COX-2, coincident with reduction of the unmodified HA-COX-2 (Fig. 3*G* and *SI Appendix*, Fig. S4*K*).

In addition to the dramatic increase in COX-2 protein levels in *HECTD4*-KD cells grown in suspension, we also observed a modest increase in *COX-2* mRNA under these conditions (*SI Appendix*, Fig. S5*A*). Two of the 25 candidate HECTD4 target proteins identified in our proteomic screen (Fig. 3 *D* and *E*), MEK1 (MAP2K1) and MKK7 (MAP2K7) (20, 21), are mitogen-activated kinase (MAPK) family members known to activate transcription factors c-Jun, ATF2, and Elk1, which function to induce *COX-2* gene expression (*SI Appendix*, Fig. S5*B*). In *HECTD4*-KD cells, the MEK1 inhibitor trametinib does not alter *COX-2* mRNA or protein levels, despite effectively inhibiting its direct target ERK1/2 (*SI Appendix*, Fig. S5 *C*–*E*). By contrast, either short-interfering RNA (siRNA)-mediated suppression of MKK7 or treatment with the MKK7 inhibitor 5Z-7-oxozeaenol (22) reverses the *HECTD4*-KD-dependent increase in *COX-2* mRNA (Fig. 3*H* and *SI Appendix*, Fig. S5*F*). Importantly, in *HECTD4-MKK7* double KD cells, COX-2 protein remains elevated, pointing to direct protein stabilization by loss of HECTD4 (Fig. 3*I*). To confirm that MKK7 itself is an HECTD4 target protein, we demonstrated its accumulation at the protein level (*SI Appendix*, Fig. S5*G*) in *HECTD4*-KD cells, without changes in *MKK7* mRNA (*SI Appendix*, Fig. S5*H*). ALFA-tag pull-down of endogenous HECTD4 also mediates ubiquitination of in vitro translated HA-tagged MKK7 (Fig. 3*J* and *SI Appendix*, Fig. S4*K*).

Thus, HECTD4-mediated ubiquitination regulates COX-2 expression both directly and indirectly, through ubiquitin-mediated degradation of COX-2 protein itself and through targeting of its upstream transcriptional regulator MKK7, respectively.

### HECTD4 Modulation of COX-2-Dependent Tumorigenesis and Metastasis.

**Fig. 4.**
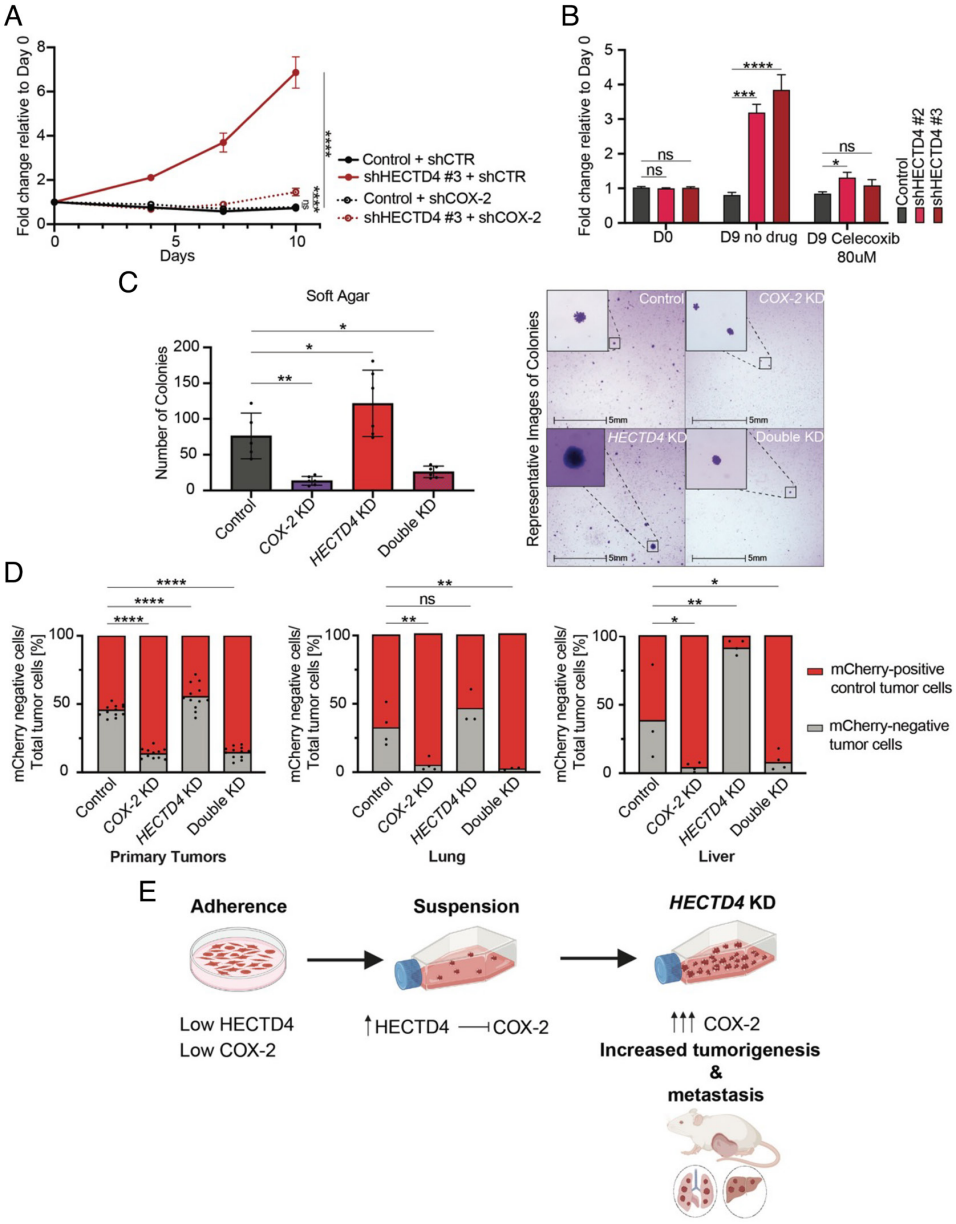
HECTD4 modulation of COX-2-dependent tumorigenesis and metastasis. (*A*) Depletion of *HECTD4* increases cell proliferation under anchorage-independent, suspension conditions (ultralow-adherent culture dish) compared to scrambled control. Depletion of *COX-2* in the *HECTD4*-depleted cells reverts this phenotype. Cell viability and proliferation measured by CellTiter Glo luminescence. Error bars represent mean ± SD. Significance was calculated using with two-way ANOVA test, with Tukey’s multiple comparison test (Day 10). The mRNA levels of *PP1A, HECTD4,* and *COX-2* in the cells used for this experiment are shown in *SI Appendix*, Fig. S6*A*. Only the cells harboring the control and shHECTD4 #3 shRNAs are shown in this figure. Cells with the control and shHECTD4 #1 shRNAs are shown in *SI Appendix*, Fig. S6*B*. (*B*) *HECTD4*-KD cell proliferation is increased under anchorage-independent, suspension conditions (ultralow-adherent culture dish) compared to scrambled control which remain stable after 9 d without treatment. Addition of celecoxib (80 umol/L) reverses the proliferation advantage of *HECTD4*-KD cells. Cell viability measured by CellTiter Glo luminescence upon celecoxib treatment. Error bars represent mean ± SD. Significance was calculated using the one-way ANOVA test, with Dunnett’s multiple comparison test. The baseline mRNA levels of *PP1A, HECTD4,* and *COX-2* in the cells used for this experiment are shown in *SI Appendix*, Fig. S5*C*. The same cells were used in the experiments in *SI Appendix*, Fig. S5*D*. (*C*) Four different groups (scramble control, *COX-2*-KD, *HECTD4*-KD, and double KD) of single MDA-MB-231 cells were seeded on 6-well plates coated with soft agar (10,000 cells/well). After 2 wk, the colonies were stained with crystal violet and counted using the HALO software. Error bars represent mean ± SD. Significance was calculated using the one-way ANOVA test, with Dunnett’s multiple comparison test (*Left*). Representative images of colonies from this experiment (*Right*). The mRNA levels of *HECTD4* and *COX-2* in the cells used for this experiment are shown in *SI Appendix*, Fig. S6*D*. The same cells were used in the in vivo mixing experiment in Fig. 4*D*. (*D*) Fraction of the four GFP+/mCherry- cell mixing conditions: i. GFP-shControl (M1-M6), ii. GFP-*COX-2*-KD (M7-M12), iii. GFP-*HECTD4*-KD (M13-M18), and iv. GFP-Double-KD (M19-M24) [%] (n = 6 mice per group). Primary tumors were bilaterally injected into the mammary fat pads of each mice (see *Materials and Methods* for more detail) and the fraction of tagged cells arising within each mixed tumor cell populations was calculated using flow cytometry, as depicted in the schematic *SI Appendix*, Fig. S7*A*. Bars represent the percentage of red (GFP-mCherry-shControl, shown in red) and green (i. GFP-shControl, ii. GFP-*COX-2*-KD, iii. GFP-*HECTD4*-KD, and iv. GFP-Double-KD, shown in gray) cells in the primary (*Left*) and metastatic tumors (Middle-Lungs, Right-Liver). Dots represent the ratio of mCherry-negative cells (manipulated) compared with m-Cherry-tagged (control) within each tumor. Significance was calculated using the one-way ANOVA test, with Dunnett’s multiple comparison test. The mRNA levels of *HECTD4* and *COX-2* in the cells used for this experiment are shown in *SI Appendix*, Fig. S6*D*. The same cells were used in the soft agar experiment in Fig. 4*C*. The day 0 (in vitro) injection ratios of the cells used in this experiment are shown in *SI Appendix*, Fig. S7*B*. Representative images of the primary tumors, livers, and the lungs are presented in *SI Appendix*, Fig. S7 *C*–*E*, respectively. The volumes of the primary tumors are shown in *SI Appendix*, Fig. S7*F*. (*E*) Schematic model demonstrating the proposed function of HECTD4 as a suppressor of tumorigenesis and metastasis, through its regulation of COX-2. HECTD4 and COX-2 levels are undetectable under adherent cell culture. The levels of both proteins rise under anchorage-independent conditions, and suppression of *HECTD4* results in massive COX-2 protein accumulation. High COX-2 levels in the setting of HECTD4 repression are associated with increased proliferation in vitro, as well as increased tumorigenesis and metastasis in vivo. (*P < 0.05, **P < 0.01, ***P < 0.001, and ****P < 0.0001).

Given the dramatically increased COX-2 protein expression upon *HECTD4* depletion in epithelial cells that lose matrix attachment, we sought to determine whether the tumorigenic phenotype evident in *HECTD4*-KD cells is dependent upon COX-2. Indeed, shRNA-mediated suppression of COX-2 abrogates the increased proliferation of *HECTD4*-KD cells when these are grown under anchorage-independent conditions (Fig. 4*A* and *SI Appendix*, Fig. S6 *A*–*C*) as does the selective COX-2 inhibitor, celecoxib (Fig. 4*B* and *SI Appendix*, Fig. S5*C*). Remarkably, we find that soft agar colony formation itself is dependent upon COX-2 activity, suggesting an essential contribution of COX-2 for cell proliferation under anchorage-independent states. Consistent with its upstream regulation of COX-2, *HECTD4*-KD fails to rescue the *COX-2*-KD phenotype (Fig. 4 *C*, *Left*, *Right* and *SI Appendix*, Fig. S6*D*).

To test whether the in vivo tumorigenic phenotype of *HECTD4*-KD MDA-MB-231 cells is also dependent on COX-2, we set up a cell mixing experiment analogous to that described above (Fig. 2*B* and *SI Appendix*, Fig. S7*A*). Using pair-wise 1:1 mixing experiments with control cells, we tested the tumorigenic contributions of *COX-2*-KD, *HECTD4*-KD, and the double *HECTD4/COX-2*-KD, using tagged GFP and mCherry markers (*SI Appendix*, Figs. S6*D* and S7 *A*–*F*). As with our previous mixing experiment, primary tumors were harvested on day 25 after orthotopic inoculation, and lungs and liver were collected on day 54 to evaluate metastatic growth following removal of the primary tumor. FACS analysis of primary tumors and metastases confirms the increased tumorigenesis of *HECTD4*-KD cells (Fig. 4*D*). *COX-2*-KD cell populations are profoundly depleted from tumors, consistent with the tumor-suppressing effect of COX-2 inhibition (23, 24). In the double KD, the simultaneous suppression of COX-2 completely abrogates the increased tumorigenic effect of *HECTD4*-KD (Fig. 4 *D*, *Left*). Similar results are observed in the metastatic lung and liver lesions, where *COX-2*-KD alone dramatically suppresses metastatic dissemination and the double KD completely reverses the growth advantage conferred by the *HECTD4*-KD (Fig. 4 *D* *Middle*, *Right*). Taken together, the tumor and metastasis suppressor effect of HECTD4 is dependent on suppressing COX-2 activity.

## Discussion

We demonstrate a tumor and metastasis suppressor role for HECTD4, a previously uncharacterized member of the HECT family of E3 ubiquitin ligases. Our initial CRISPR-i screen scored HECTD4 as a suppressor of metastasis initiation by breast cancer patient-derived CTCs, and our follow-up analyses point to a critical effect in anchorage-independent cell proliferation, with COX-2 as a major degradation target. Together, these observations suggest a potentially critical role for this prostaglandin-synthetic pathway in the survival of epithelial cancer cells in the circulation (Fig. 4*E*).

Among the best-studied protein degradation pathways implicated in tumorigenesis are von Hippel–Lindau targeting of HIF, MDM2 degradation of TP53, FBXW7 destabilization of MYC and SKP2 degradation of p27/CDKN1B (25). Furthermore, several different deubiquitinases have also played a role in cancer, such as USP11 in the regulation of the estrogen receptor in breast cancer, USP15 in TGF-β signaling in glioblastoma and USP26 in androgen receptor regulation (26–29). Our initial loss-of-function in vivo CRISPR-i screen identified previously characterized high-ranking hits in the ubiquitin-proteasomal and lysosomal degradation pathways, *FBXL6, WWP1, UHRF2, KCMF1, JADE2,* and *RNF121.* However, HECTD4 had remained uncharacterized, with no known function or substrate, leading us to further pursue its role in tumorigenesis and metastasis. Interestingly, within the HECT gene family, not all proteins mediate proteolytic ubiquitination. HERC2 promotes BRCA1 degradation, and WWP1 promotes ubiquitination and degradation of p27, ErbB4, and other substrates, but SMURF1-mediated ubiquitination stabilizes estrogen receptor α, while HECTD3 has opposing ubiquitination effects, degrading caspase 8, while stabilizing the NFkB regulator MATL1 (13). Our studies of the previously uncharacterized HECT gene family member HECTD4 thus establish its catalytic functions as a degradative E3 ubiquitin ligase, with COX-2 as a major degradation target. We note that we previously conducted a CRISPR-activation (CRISPR-a) screen in breast cancer CTCs, nominating multiple regulators of protein translation, including core structural components of the ribosome, as enhancers of CTC-mediated tumorigenesis (4). Together, these loss of function and gain of function CRISPR screens highlight the relevance of protein synthesis and degradation as regulatory factors in tumorigenesis and metastasis.

A role for the ubiquitin ligase HECTD4 in cancer is supported by clinical databases indicating a correlation between reduced HECTD4 expression in breast cancer and an adverse progression-free survival (16). The most striking characteristic, drawn from our experimental models, is its unique contribution to anchorage-independent proliferation. Indeed, HECTD4 is expressed at low levels in cultured cells grown under standard 2D matrix attachment conditions, but it is induced as these cells lose their anchorage dependence. It is only under anchorage-independent culture conditions that *HECTD4* knockdown mediates an effect on cell proliferation. Anoikis is thought to be the predominant form of cell death facing epithelial cancer cells that intravasate into the bloodstream, and its suppression is likely to contribute to the ability of CTCs to survive in circulation and ultimately give rise to metastatic lesions. In this context, the targeting of COX-2 by HECTD4, both through direct protein ubiquitination and through suppression of its regulatory kinase MKK7, highlights a potentially important role for this prostaglandin synthetic pathway in the survival of metastatic precursors in circulation.

COX-2 is the rate-limiting synthetic enzyme in the synthesis of the prostaglandin PGE2, mediating an array of protumorigenic inflammatory signals (5). These include secretion of growth factors mediating angiogenesis, cell proliferation, apoptotic resistance, and cell invasion. The role of COX-2 in enhancing cancer has been primarily derived from seminal epidemiological studies of chemoprevention through administration of nonsteroidal anti-inflammatory drugs (NSAID) (7, 30, 31). These studies demonstrated a potent suppression of cancer initiation across colorectal and other cancer types. However, the broad utility of NSAIDs and selective COX-2 inhibitors as chemopreventive agents is negated by their concurrent activation of thrombotic pathways, resulting in increased cardiovascular events in individuals who do not have cancer (7, 32). Nonetheless, additional studies have also demonstrated a beneficial effect of COX-2 inhibition in the setting of metastatic progression (9), which may present a different risk/benefit calculation, depending on the relative risk of cancer recurrence versus cardiovascular complication.

In multiple mouse models, COX-2 inhibition prevents or reduces the development of metastasis from breast, colorectal, and lung tumors (33, 34). While clinical studies of metastatic suppression are challenging to undertake, one meta-analysis indicates that daily aspirin use is associated with a reduced risk of presenting with metastatic adenocarcinoma, or of developing metastases following treatment of a primary cancer (10). In a recent study, including celecoxib as an adjuvant to treat stage III colon cancer was associated with improved disease-free survival and overall survival (35). However, another study found no effect of high-dose aspirin in preventing early recurrence of breast cancer (11). Of note, the combination of short-term COX-2 inhibition with chemotherapy or other modalities for the treatment of established metastatic disease has proven ineffective. Taken all together, translating the striking effect of COX-2 inhibition in preventing cancer metastasis in mouse models to human clinical studies will require careful consideration of metastatic risk stratification, as well as drug dosage, duration of treatment, and appropriate endpoints. In this context, the role of COX-2 inhibition in the prevention of blood-borne metastasis may impact the optimal design of clinical studies.

Our observations point to a distinct mechanism whereby COX-2 may mediate its prometastatic effects. While most studies have focused on the inflammatory output of prostaglandins in cancer initiation, the specific anchorage-independent growth context in which HECTD4 regulates COX-2 expression indicates a distinct role in sustaining epithelial cancer cells as they circulate in the bloodstream. Consistent with such a cell-autonomous and non-inflammation-mediated mechanism, COX-2 has been reported to enhance expression of E-cadherin under 3D culture conditions, thereby sustaining reattachment of suspended cells onto matrix and enhancing tumorigenesis (36). Thus, suppressing the ability of circulating cancer cells to survive in the bloodstream and initiate metastatic growth through the HECTD4–COX-2 axis reported here highlights a therapeutic opportunity, which may warrant clinical investigation of metastasis prevention in carefully selected clinical contexts.

## Materials and Methods

All materials and methods are described in detail in *SI Appendix*. The animal experiments were conducted in accordance with institutional ethical guidelines and approved under Institutional Animal Care and Use Committee protocol #2010N000006. Target sequences for the shRNA constructs are listed in *SI Appendix*, Table S1. Prime-editing sequences for ALFA-tagging are in *SI Appendix*, Table S2. The sequences of the primers for qRT-PCRs are provided in *SI Appendix*, Table S3. The primary antibodies used for western blotting are listed in the *SI Appendix*, Table S4. Methods for cell culture, CRISPR experiments, plasmid construction, generation of knockdown cell lines, proliferation and colony assays, RNA extraction and qPCR, western blotting, proteomics, ubiquitination assays, clinical dataset analysis, and all mouse experiments are detailed in *SI Appendix*.

### Statistical Analysis.

GraphPad Prism software (version 10.2.2) was used to generate graphs and to perform the corresponding statistical tests indicated in the figure legends.

### Illustrations.

Illustrations were created with BioRender.com.

## Supplementary Material

Appendix 01 (PDF)

## Data Availability

All mass spectrometry RAW data can be accessed through the MassIVE data repository (massive.ucsd.edu) under the accession number MSV000096318 (37). All other data are included in the manuscript and/or *SI Appendix*.
