# Supplementary material for: The E3 ligase HECTD4 regulates COX-2-dependent tumor progression and metastasis
Source: Proc Natl Acad Sci U S A. 2025 Aug 6;122(32):e2425621122. doi: 10.1073/pnas.2425621122 (PMC12358835; doi:10.1073/pnas.2425621122)
Supplement: Supplementary file 1 — Appendix 01 (PDF) [file pnas.2425621122.sapp.pdf]

PNAS

Supporting Information for

The E3 ligase HECTD4 regulates COX-2 dependent tumor progression and metastasis

Joanna A. Vuille[a,b,1], Cem Tanriover[a,1], Ezgi Antmen[a], Douglas S. Micalizzi[a], Richard Y. Ebright[a], Sambhavi Animesh[a], Robert Morris[a], Soroush Hajizadeh[a], Zachary J. Nicholson[a], Hunter C. Russell[a], Eric F. Zaniewski[a], Ben S. Wittner[a], Ben K. Wesley[a], Ji Eun Kwak[a], Julian Grünewald[c,d], Regan N. Szalay[c,d], Douglas B. Fox[a], Min Yang[a,e], J. Keith Joung[a,d,f], Doga C. Gulhan[a], Andrew E.H. Elia[a,g], Wilhelm Haas[a], Eugene Oh[a], Shyamala Maheswaran[a,2], Daniel A. Haber[a,e,2]

[1]J.A.V. and C.T. contributed equally to this work.

[2] To whom correspondence may be addressed. Email: ✉ maheswaran@helix.mgh.haravard.edu or ✉ dhaber@mgh.harvard.edu.

This PDF file includes:

Figures S1 to S7
Tables S1 to S4
SI References

Supporting Information Text

Materials and Methods

Cell culture

For the initial CRISPR screen, the circulating tumor cell (CTC) line Brx-142 was cultured in suspension as previously described (1-3). Brx-142 cells were authenticated by RNA-seq and DNA-seq.

Human mammary cancer cell lines MDA-MB-231, HCC1143, BT-549 and MDA-MB-468 cells were all obtained from ATCC and derived from female patients. Cells were cultured on adherent plates (Corning) at 37 °C and 5% CO2 in humidified culture incubators. MDA-MB-231 and MDA-MB-468 cells were grown in DMEM high glucose medium (Gibco) supplemented with 10% fetal bovine serum (FBS) (Gibco) and 1X penicillin/streptomycin (Pen/Strep) (Gibco). HCC1143 and BT-549 cells were cultured in RPMI-1640 with GlutaMAX (Gibco) supplemented with 10% FBS (Gibco) and 1X Pen/Strep (Gibco). To assess HECTD4, COX-2 and MKK7 levels in suspension, cells were seeded in anchorage-independent, ultra-low attachment plates (Corning) and cultured for 3 to 5 days prior to harvest. The cell lines used in this study were routinely tested for mycoplasma every 2 months using the Mycoalert kit (Lonza).

Lentiviral production and infection of the CTCs with the CRISPR inactivation library

Lentivirus generation and the infection of Brx-142 with the CRISPR inactivation library was conducted as as previously described (3). Briefly, HEK293T cells were transfected with psPAX2 (Addgene #12260), pMD2.G (Addgene #12259), and pooled library plasmid with the Lipofectamine 2000 and the PLUS reagent. Viral supernatants were collected 48h post-transfection. The CTCs were infected with the lentivirus and were selected with puromycin.

CRISPR inactivation screen – mice injection and organ harvest

Mice injections and the harvesting of the organs were performed as previously described (3). Brx-142 cells stably expressing GFP, luciferase, and KRAB-dCas9 (Addgene #96918) were transduced with the human CRISPR Inhibition Pooled Library (Dolcetto pool A, Addgene #92385) (4), at a multiplicity of infection (MOI) of 0.3, and selected with puromycin to maintain a 300-350 cells per sgRNA coverage. In parallel, plasmid DNA (pDNA) used for viral transduction was isolated as the input reference distribution of guides. Eight female NSG (NOD. CgPrkscid Il2rgtm1Wjl/SzJ) mice were injected via tail vein with $3x10^6$ cells each and implanted with 90-day release estrogen pellets. After three months, lungs were harvested and processed for genomic DNA extraction. sgRNA representation was analyzed by PCR amplification performed by the Broad Institute Screening Platform, and products were sequenced on the Illumina MiSeq platform.

CRSIPR inactivation Screen - Analysis

Analysis for the CRISPR inactivation screen was performed as previously described (3). Guide counts from all 8 mice were aggregated and were normalized to the total read counts. Guide distribution was compared

to the plasmid input distribution of guides, resulting in a fold change value for each guide. Guides with input counts below 100 were excluded. For each gene, the most enriched guide was identified, and genes were rank-ordered based on fold enrichment, with rank 1 representing the highest enrichment.

Lentiviral Packaging and Stable Cell Line Generation

The following plasmids were used for lentiviral packaging: pLKO.1-puro lentiviral constructs expressing shRNA against human *HECTD4* and *COX-2*, respectively, were obtained from the MGH Molecular profiling laboratory (MPL) at the MGH Cancer Center. A scramble shRNA control vector (Addgene #136035) was used as a negative control. For stable expression of GFP and luciferase, a GFP-luciferase construct was used. Additional vectors included a luciferase-Blasticidin construct (Addgene #19166) and an mCherry expression plasmid (Addgene #176016). Lentiviral particles were produced by transfecting HEK293T cells with the relevant transfer plasmids, together with the pMD2.G (Addgene #12259) and psPAX2 (Addgene #12260) packaging plasmids, using Lipofectamine 2000 reagent (Invitrogen). At 48-72 h post-transfection, supernatants containing lentiviral particles were harvested, filtered through a 0.45 μm PVDF filter, aliquoted, and stored at −80 °C. For transduction, viral supernatants were added to MDA-MB-231, HCC1143, BT-549, and MDA-MB-468 cells in the presence of polybrene (8 μg/mL; Santa Cruz) and incubated overnight. Following infection, cells were selected with the appropriate antibiotics: Puromycin (2 μg/mL) for 72 hours or Blasticidin (3 μg/mL) for 5-6 days.

The *HECTD4* and *COX-2* mRNA sequences targeted with lentiviral shRNA clones are listed in **table S1**.

Transient Knockdown of MKK7 and COX-2, using siRNA

For transient knockdown of *COX-2* or *MKK7*, MDA-MB-231 in monolayer cultures were treated with a SMARTpool comprised of 4 different siRNAs targeting COX-2 (Horizon Discovery # L-004557-00-0005), MKK7 (Horizon Discovery # L-004016-00-0005) or control (non-targeting pool, Horizon Discovery # D-001810-10-05). The cells were seeded into 6-well plates (1.5 x 10^5 cells/well), and treated with 0.625 μL of siRNA 20 μM, mixed with 6 μL of lipofectamine RNAiMAX (Thermo Scientific #13778075) in reduced serum medium (Opti-MEM, Thermo Scientific #31985062), according to the manufacturer's instructions. The cells were placed in suspension after 24 hours and/or analyzed at the indicated time point.

Orthotopic tumorigenesis using homogenous cell populations

GFP+/luciferase+ MDA-MB-231 were transduced with either the scramble control vector or shHECTD4 #1 and fully selected with puromycin. For the orthotopic injections, 2x10^5 cells from each group were resuspended in 100 μL of a 1:1 mixture with Growth Factor Reduced Matrigel (Corning #354230). The cell suspension (100 μL total volume) was injected into the right inferior mammary fat pad of 4 female NSG mice for the control and *HECTD4*-KD groups, respectively. On day 34, the mice were sacrificed, and the primary tumors were harvested. Tumor weight and volume were measured at the time of sacrifice.

Mouse tumor cell mixing experiments for orthotopic inoculation and metastasis measurements (HECTD4-KD)

GFP+/luciferase+/ mCherry- MDA-MB-231 were infected with the scramble control vector (GFP-shControl; *HECTD4*-WT) and GFP+/luciferase+/ mCherry+ MDA-MB-231 were infected with shHECTD4 (sh #1) (GFP-mCherry-*HECTD4*-KD) or with the scramble control vector (GFP-mCherry-shControl; *HECTD4*-WT). Cells were fully selected with puromycin (2µg/mL). Two different 1:1 mixing cell populations were generated: 1. GFP-shControl cells were mixed with GFP-mCherry-shControl cells, and separately, 2. GFP-shControl cells were mixed with GFP-mCherry-*HECTD4*-KD cells. An aliquot of each mix was saved for FACS analysis to determine the initial injection ratios. For the injections, $2x10^5$ cells of the mixes were further combined into 1:1 Growth Factor Reduced Matrigel (Corning #354230). The cell media (100 µL total volume) was orthotopically injected into the right inferior mammary fat pad of 4 female NSG mice for the control mix and 5 mice for the *HECTD4*-KD mix. Metastatic growth was monitored weekly via in vivo imaging using the IVIS Lumina II (PerkinElmer) following intraperitoneal injection of D-luciferin (Sigma), administered 5 minutes prior to imaging. Survival surgery was perfomed at day 36 to harvest the primary tumors. At day 62-65, the mice were sacrificed, and lungs and livers were harvested. Fluorescent pictures of the organs were taken with an MVX10 Macro Zoom Fluorescence microscope (Olympus) with GFP and mCherry filter. The organs were mechanically dissociated, followed by a biological digestion with collagenase/hyaluronidase (StemCell Technologies Inc. # 07912) in DMEM by rotation for 3 hours at 37°C. Digested lysates were cleared by centrifugation and washed in PBS. Single cells were filtered through a mesh strainer and analyzed using an BD LSRFortessa X-20 flow cytometer (BD Bioscience) and a SH800S (Sony sorter). Cytometry data were processed with FlowJo software (version 10.8.1). One liver sample in the *HECTD4*-KD group was excluded from the final analysis due to tissue and cellular damage as a result of the digestion process. In total, n = 4 for WT and n = 5 for *HECTD4*-KD for primary tumors; n = 4 for WT and n = 5 for *HECTD4*-KD for the lungs; n = 4 for WT and n = 4 for *HECTD4*-KD for the livers.

Mouse Tail Vein Injections (HECTD4-KD)

Luciferase+ MDA-MB-468 cells harboring either the scramble control vector (M1-M6) or shHECTD4 #2 (M7-M12) were used for this tail vein metastasis experiment. For each injection, $5x10^5$ cells were resuspended in 100 µL of sterile PBS and administered via the lateral tail vein of immunosuppressed NSG mice (n = 6 per group). Metastatic tumor burden was monitored weekly by in vivo bioluminescence imaging using the AMI HT Imaging System (Spectral Instruments Imaging) following intraperitoneal injection of D-luciferin (Sigma), administered 5 minutes prior to imaging. On day 28, the final images were acquired, and the mice were sacrificed for analysis. Regions of interest (ROIs) encompassing the lung fields were defined using Aura Imaging Software (Spectral Instruments Imaging) to quantify total flux (photons/sec). Radiance scale bars indicating minimum and maximum signal intensities were included in each image. Quantified total flux values from the lung regions of individual mice were plotted and analyzed for statistical significance.

Mouse tumor cell mixing experiment for orthotopic tumorigenesis and metastasis (HECTD4-KD plus COX-2-KD)

GFP+/luciferase+ MDA-MB-231 cells were infected with the mCherry and the scramble shRNA vector as control (GFP-mCherry-shControl; *HECTD4*-WT and *COX-2*-WT). This control group was mixed separately with each of the following four groups (1:1 ratio for each individual mixing experiments):

i) Control. GFP+/luciferase+/mCherry - MDA-MB-231 cells infected with scramble control vector (GFP-shControl; *HECTD4*-WT and *COX-2* WT) (M1-M6);

ii) *COX-2*-KD. GFP+/luciferase+/mCherry - MDA-MB-231 cells infected with shCOX-2 (sh #1 and sh #2 mixed in a 1:1 ratio) (GFP-*COX-2*-KD; *HECTD4*-WT and *COX-2* KD) (M7-M12);

iii) *HECTD4*-KD. GFP+/luciferase+/mCherry - MDA-MB-231 cells infected with shHECTD4 (sh #1, sh #2 and sh #3 mixed with a 1:1:1 ratio) (GFP-*HECTD4*-KD; *HECTD4*-KD and *COX-2* WT) (M13-M18); and

iv) *Double-KD (HECTD4-KD* plus *COX-2-KD)*. GFP+/luciferase+/mCherry - MDA-MB-231 cells infected with shHECTD4 (sh #1, sh #2 and sh #3 mixed with a 1:1:1 ratio) and with shCOX-2 (sh #1 and sh #2 mixed in a 1:1 ratio) (GFP-Double-KD; *HECTD4*-KD and *COX-2* KD) (M19-M24).

Cells were fully selected with puromycin (2µg/mL). GFP-mCherry-shControl cells were mixed with i. (GFP shControl), ii. (GFP-*COX-2*-KD), iii. (GFP-*HECTD4*-KD) and iv. (GFP-Double-KD) at a ratio of 1:1. An aliquot of all four mixes was saved for FACS analysis to determine the initial injection ratios. For the injections, $2x10^5$ cells of the mixes were further combined into 1:1 Growth Factor Reduced Matrigel (Corning #354230). The cell-matrigel mixes (100 µL total volume) were injected into both the right and left inferior mammary fat pad of female NSG mice (n=6 mice per group; total of 24 mice). Primary tumor and metastatic growth were measured weekly via in vivo bioluminescence imaging using the AMI HT Imaging System (Spectral Instruments Imaging) following intraperitoneal injection of D-luciferin (Sigma), administered 5 minutes prior to imaging. Survival surgeries were performed on four out of the six mice in each group at days 24-25 to harvest the primary tumors. Two mice from each group were sacrificed and their primary tumors as well as lungs and livers were collected to assess for early metastasis development in the these organs. The volumes of all primary tumors were measured at this time. At day 54-55, the remaining four mice per group were sacrificed, and their lungs and livers were harvested. Photographs of the primary tumors, and of lungs and livers were taken at the time of tissue resection. The organs were mechanically dissociated, followed by biological digestion with collagenase/hyaluronidase (StemCell Technologies Inc. # 07912) in DMEM by rotation for 3 hours at 37°C. Digested lysates was cleared by centrifugation and rinsed in PBS. Single cells were filtered through a mesh strainer and analyzed using an BD LSRFortessa X-20 flow cytometer (BD Bioscience) and a SH800S (Sony sorter). Cytometry data were processed with FlowJo software (version 10.8.1). 15 out of the 16 primary tumors from eight mice sacrificed at days 24-25 were included in the final analysis (two mice per group, one primary tumor sample was excluded from the *COX-2* KD group due to tissue and cellular damage during digestion). Lungs and livers from these mice showed

no metastases at this early time point. Of the 16 mice analyzed at the terminal time point for scoring of metastases (4 mice per group), one primary tumor sample in the Double KD (*HECTD4*-KD plus *COX-2*-KD) group, one lung sample in the *HECTD4*-KD group, one lung sample in the Double KD group and one liver sample in the WT group were excluded from the final analysis due to tissue and cellular damage as a result of the digestion process. In total, for analysis of primary orthotopic tumors, n = 12 for WT, n = 11 for COX-2-KD, n = 12 for *HECTD4*-KD and n = 12 for Double KD (*HECTD4*-KD plus *COX-2*-KD). For analysis of lung metastases, n = 4 for WT, n = 4 for *COX-2*-KD, n = 3 for *HECTD4*-KD and n = 3 for Double KD (*HECTD4*-KD plus *COX-2*-KD). For analysis of liver metastases, n = 3 for WT, n = 4 for *COX-2*-KD, n = 4 for *HECTD4*-KD and n = 4 for Double KD (*HECTD4*-KD plus *COX-2*-KD).

Proliferation assays

Metabolic activity was assessed using the CellTiter-Glo Luminescent Cell Viability Assay (Promega), according to the manufacturer's instructions. Cells were seeded at a density of 3,000 cells per well in either 3 or 6 replicates into 96-well flat-bottom, tissue culture treated microplates (for adherent conditions) or 96-well clear ultra-low attachment microplates (for anchorage-independent suspension conditions) (Corning). Plates were incubated for the indicated time periods prior to luminescence reading. Luminescence was measured using a SpectraMax M5 plate reader (Molecular Devices). All values were normalized to the corresponding day of plating and further normalized to the control condition within each plate.

Colony Formation Assay in Soft agar

A total of 10,000 cells were suspended in assay medium containing 0.3% agarose and seeded onto a pre-solidified base layer of 0.6% agarose in six-well plates. The plates were allowed to solidify at 4°C and then incubated at 37°C. Cultures were maintained for 27 days and fed weekly with 1 mL of standard growth medium. At the endpoint, colonies were fixed and stained with 500 µL of crystal violet staining solution (0.05% crystal violet, 1% formaldehyde, 1% methanol) for 30 minutes, followed by PBS washes. Colonies larger than 50 µm in diameter were imaged using the MVX10 Macro Zoom Fluorescence Microscope (Olympus) under brightfield and quantified using HALO image analysis software.

Prime-editing ALFA-tagging of endogenous HECTD4

MDA-MB-231 cells were seeded at a density of 30,000 cells per well into 24-well flat-bottom tissue culture plates (Corning). Twenty-four hours post-seeding, cells were transfected with 4.5 µg of the PE2 plasmid (Addgene #132775), 1.5 µg of pegRNA, and 500 ng of ngRNA plasmid per well. Transfections were performed using TransIT-X2 transfection reagent (Mirus, MIR #6004) at a volume of 0.3 µL per transfection. The sequences used are listed in **table S2.**

RNA extraction, cDNA synthesis and quantitative real-time PCR

RNA extracted from cultured breast cancer cells was prepared using the RNeasy Mini kit (QIAGEN) with DNase I digestion on the column according to the manufacturer's instruction. cDNA was synthesized from 100-500 ng RNA using SuperScript IV VILO Master Mix (Invitrogen # 11756050). qPCR was performed using the primers listed in **table S3**. The reactions were run by QuantumStudio 5 instrument (ThermoFisher Scientific). Each reaction was performed in 3 or 6 replicates and fold expression change was calculated using the comparative DDCt method, normalized to PP1A or GAPDH endogenous controls.

Protein cell lysate Preparation and Western blot

Cells were washed in ice-cold PBS and lysed either in RIPA buffer (Thermo Scientific #NC9484499) or Triton X-100 lysis buffer (Thermo Scientific #J62289.AK) containing a cocktail protease inhibitor (HaltTM Protease Inhibitor Cocktail, Thermo Scientific #78429). The lysates were incubated on ice for 20 minutes and cleared by centrifugation at 4 °C for 10 minutes at 13'000 RPM. The supernatants were used for protein sample preparation. Protein concentration was determined using the DC protein assay (Bio-Rad, # 5000122). The samples were diluted in PBS together with loading and reducing agents and heated at 95°C for 5 min. Equal amounts of protein (between 10 ug and 20 ug) were separated by SDS-PAGE on 4-15% (Bio-Rad, #5671084), 4-20% (Bio-Rad, #4561093), or 7.5% (Bio-Rad, #4561023) polyacrylamide gradient gels and transferred onto nitrocellulose membranes using the iBlot 2 Gel Transfer Device (Thermo Fisher). After blocking with PBS-T with 5% milk for 1 hour at room temperature, membranes were incubated with primary antibodies overnight at 4 °C at the recommended concentrations. The primary antibodies are listed in the **table S4.** After three 5-minute washes in PBS-T, the membranes were incubated with HRP-conjugated secondary antibodies (1:10,000; Bio-Rad, #5196–2504) for 1 hour at room temperature. Following another round of three 5-minute PBS-T washes, signal was developed using enhanced chemiluminescence substrate (Bio-Rad, #1705062) and visualized using the ChemiDoc MP Imaging System (Bio-Rad, #1705061).

Proteomics: Cell lysis, protein digestion, and TMT labeling

HECTD4-KD (shHECTD4 #3, n=3) and scrambled control MDA-MB-231 (n=3) cells were seeded in ultra-low adherent plates (Corning) for four days. The proteasome inhibitor MG132 (Selleck #S2619, final concentration of 20 µM) and lysosomal inhibitor (Bafilomycin A1: Sigma Aldrich #B1793, final concentration of 20 nM) were added to the cells, at the indicated concentrations, for 6 hours. Cell pellets were processed as previously described (5, 6). Briefly, cell pellets were lysed with 1000 µL of lysis buffer (75 mM NaCl, 3 % SDS, 1 mM NaF, 1 mM beta-glycerophosphate, 1 mM sodium orthovanadate, 10 mM sodium pyrophosphate, 1 mM PMSF and 1X Roche Complete Mini EDTA free protease inhibitors in 50 mM EPPS, pH 8.5). Proteins were then reduced with 5 mM DTT at 56°C for 30 min and alkylated with 15 mM iodoacetamide in the dark at room temperature for 20 min. Tricholoracetic acid (TCA) was used to precipitate the reduced and alkylated proteins. Proteins were solubilized in 1M urea in 50mM EPPS, ph 8.5 and digested in a two-step process (overnight digestion at room temperature with 1 µg/µL of Lys-C

(Wako) followed by six hours of digestion with trypsin (sequencing grade, Promega) at a final concentration of 1 ng/μL for 6 hr at 37 ˚C). The digest was acidified with 10 % TFA and peptides were desalted on C18 solid-phase extraction (SPE) (Sep-Pak, Waters) columns. The concentration of the desalted peptide solutions was measured with a BCA assay, and peptides were aliquoted keeping 1950ug for the diGly proteomics and 100ug for total proteome. The samples were dried under vacuum and stored at -80 ˚C until they were labeled with TMT reagents. For total proteomics, TMT labeling was performed with 18-plex tandem mass tag (TMT) reagents (Thermo Scientific) as described below (5). The TMT labeled peptides were pooled and desalted via C18 SPE and then fractionated into 24 fractions using Basic pH Reversed-Phase Liquid Chromatography (bRPLC). The individual fractions were analyzed in 3-hour runs via LC-MS2/MS3 on an Orbitrap FusionLumos mass spectrometer using the Simultaneous Precursor Selection (SPS) supported MS3 method (7, 8) .

For the diGly proteomics, the co-immunoprecipitation of KεGG-modified peptides was carried out according to manufacturer's instruction (Cell Signaling PTMScan, #59322). Briefly, the samples were resuspended in the provided binding buffer. 20uL of bead-slurry were used per condition, and the samples were incubated on an end-over-end rotator for 2 hours at 4°C. Washes were performed with the provided wash buffer, and 2 rounds of elution with IAP Elution Buffer (0.15% TFA) were performed. A second round of co-immunoprecipitation was subsequently performed with fresh bead-lysate. Eluted DiGly-peptides were combined and labeled with TMT18plex reagents (Thermo Fisher Scientific). For labeling with TMT 18 plex reagents, 25 μg of peptides were dried and resuspended in 25 μL of 200 mM EPPS (pH 8.5), 30% acetonitrile (ACN). Labeling was performed by adding 70 μg TMT reagent in anhydrous ACN and incubating at room temperature for 1 h. The reaction was stopped by addition of 5% (w/v) hydroxylamine in 200 mM EPPS (pH 8.5) to a final concentration of 0.5% hydroxylamine and incubation at room temperature for 15 min. Samples were acidified with 1% TFA, and combined. The pooled samples were desalted using Sep-Pak C18 SPE cartridges and vacuum dried. The peptides were then fractionated using Pierce™ High pH Reversed-Phase Peptide Fractionation Kit (Thermo Scientific Cat 84868) according to the manufacturer's instructions and vaccum dried. The dried fractions were resuspended in 5 % ACN/5 % formic acid and analysed with high resolution LC-MS2 on an Orbitrap FusionLumos mass spectrometer. MS2 spectra were assigned using a COMET-based in-house built proteomics analysis platform (9) allowing methionine oxidation (+15.99492 Da) and lysine ubiquitylation (+ 114.04293 Da) as a variable modification. A target-decoy database-based search was used to filter the false-discovery rate (FDR) of protein identifications of <1% (10). Peptides that matched to more than one protein were assigned to that protein containing the largest number of matched redundant peptide sequences following the law of parsimony. TMT reporter ion intensities were extracted from the MS3 spectra, selecting the most intense ion within a 0.003-*m*/*z* window centered at the predicted *m*/*z* value for each reporter ion, and spectra were used for quantification if the sum of the S/N values of all reporter ions divided by the number of analyzed channels was ≥10 and the

isolation specificity for the precursor ion was ≥0.75. Protein intensities were calculated by summing the TMT reporter ions for all peptides assigned to a protein.

The full proteome data was normalized in a two-step process, a row (protein) normalization followed by a column (sample) normalization. The row normalization involved scaling the row intensity values by the global median of all row means divided by each rows mean, adjusting each row mean to equal the global median. Similarly, the column normalization involved scaling the column intensities so that each column median would be equal the average of all sample medians. The raw diGly peptide data was normalized in an analogous procedure.

Proteomic Analysis

Pairwise Pearson correlations between replicate proteome profiles were generated and two samples, 7_Control_MG132_BafA1 (average Pearson= -0.06) and 12_KD_sh3_MG132_BafA1 (average Pearson=0.35) were removed from downstream analyses because the average correlation with their replicates was below 0.5. Differential analysis of total proteome expression levels between *HECTD4* knockdown and control sample was performed using the Limma R package (11) which uses a moderated t-statistic to generate a significance p-value for each protein. P-values were adjusted for multiple hypothesis testing by the Benjamini-Hochberg correction (12). Protein with at least 1.5 fold change and a corrected p-value < 0.25 were kept for further analysis. Differential expression analysis for the triplicate diGly data was performed as described previously for the total proteome data. DiGly peptide levels were normalized to their full protein expression levels prior to differential expression analysis. Out of the total 3447 diGly peptides quantified, normalized expression levels for 3311 peptides were generated and used in downstream analyses.

HECTD4 Short Active and Short Mutant assay

For the cloning of short active and mutant form of HECTD4, a DNA sequence coding for the aminoacids 3988 to 4428 of the *HECTD4* gene (C-terminus; short active with the regular sequence; the short mutant with the residue C4396 mutated into Alanine) in frame with the ALFA tag at its 5' end and flanked by *BamHI* and *XbaI* inside the subcloning vector pUC-GW-Amp was ordered from GENEWIZ.This fragment was subcloned by restriction (*BamHI* and *XbaI*) and ligation into the lenti-vector expression backbone plasmid pLenti-CMV-Blast-empty-(w263-1) (Addgene #125133). Sequence integrity was validated with Sanger sequencing. Lenti-vectors were prepared in 293T cells as described above. Briefly, 293T cells were transfected with the lentiviral constructs, specified above, together with pMD2.G (Addgene#12259) and psPAX2 (Addgene#12260) packaging plasmids using Lipofectamine 2000 reagent (Invitrogen). 48-72 h after transfection, culture medium (containing lentiviral particles) was collected, filtered through a 0.45µm PVDF filter, aliquoted and stored at -80°C. MDA-MB-231 transduction was carried through an overnight

incubation of lenti-vector supernatants with 8 μg/ml Polybrene (Santa Cruz). Transduced cells were selected for with 10 μg/ml Blasticidin S HCL (Gibco).

In vitro *transcription and translation*

COX-2 and MKK7 substrates were cloned under the SP6 promoter into the pCS2+N-3HA expression vector (Addgene #196653), generating the plasmids pCS2-3HA-COX-2 and pCS2-3HA-MKK7. In vitro translation was performed using rabbit reticulocyte lysate (Promega, #L2080) by incubating 3 µL (approximately 400 ng) of plasmid DNA in 25 µL of lysate for 1 hour at 30 °C. Reactions were terminated by rapid dilution in 1× PBS. The resulting HA-tagged proteins were used as substrates for the in vitro ubiquitination assays.

HECTD4 Immunoprecipitation/ purification

Cells were lysed in lysis buffer (TRIS pH 7.5 20μM, 1mM EDTA, 1mM EGTA, 150mM NaCl, 1% Triton X-100, protease and phosphatase inhibitors (ThermoFisher #78440)). Lysates were kept on ice for 20 minutes, and then cleared by centrifugation at 13,000 rpm for 15 minutes at 4°C. HECTD4 immunoprecipitation was performed using 500 μg to 1 mg of cellular lysates using magnetic beads crossed-linked to ALFA nanobody (Nanotag Biotechnologies, #N1515-L), previously rinsed in the same lysis buffer (40 μL/mg of cellular lysis). 5% of was stored as input sample.

Magnetic beads and lysis were incubated with rotation at 4°C for 2 hours. Beads were washed with ice cold lysis buffer four times. For in vitro ubiquitination assay, magnetic bead-attached HECTD4 was used as it (5uL of beads per reaction). For co-immunoprecipitation analysis, samples were eluted with urea sample buffer (120 mM Tris 6.8; 4% SDS; 4M urea; 20% glycerol, 10mM βME). Elutes were then run and analyzed by Western blots.

In vitro *Ubiquitination*

In vitro ubiquitination assays were performed in a 15 μl reaction volume: 0.25 μl of 10 μM E1 (Fisher Scientific # E304050), 1 μl of 10 μM E2 (UBE2D) (Boston Biochem # E2-622), 1 μl of 10 mg/ml ubiquitin (Boston Biochem, U-100H), 1 μl of 15 mM DTT, 1.5 μl of energy mix (150 mM creatine phosphate, 20 mM ATP, 20 mM MgCl2 , 2 mM EGTA, pH to 7.5 with KOH), 4.25 μl of 1 × PBS, 1 μl of 10 × ubiquitination assay buffer (250 mM Tris 7.5, 500 mM NaCl, and 100 mM MgCl2) and 1 μl of substrate (in vitro translated) were premixed and added to 4 μl of HECTD4-purified or empty magnetic beads (see 'HECTD4 immunoprecipitation/purification'). Reactions were performed at 30 °C with shaking for 60 min, unless noted otherwise. For the deubiquitination reaction, 0.25 μL of the de-ubiquitinating enzyme Ubiquitin Specific Peptidase 2 (USP2) (Fisher Scientific # E504050) was added to the sample following incubation for 60 min at 30 °C. The samples with USP2 were incubated an additional 30 min at 30 °C. All reactions were stopped by adding 2x urea sample buffer and resolved on SDS-acrylamide gels.

HECTD4 evolutionary tree and clinical data visualization

The evolutionary tree showing the 13 members of the HECT subfamily was developed using the Neighbor-Joining algorithm implemented in the MEGA-11 program using Poisson correction method (13). HECTD4 gene expression data (3691 normal samples, 29,376 tumors and 453 metastatic) was derived from gene chip-based studies of the Gene Expression Omnibus of the National Center for Biotechnology Information (NCBI-GEO), plotted on TNMplot.com (14). Proteomic data was obtained from the proteomic datasets of the National Cancer Institute's Clinical Proteomic Tumor Analysis Consortium (CPTAC) and National Cancer Institute's International Cancer Proteogenome Consortium (ICPC). Data was visualized by the interactive web server Cancer Proteogenomic Data Analysis Site (CProSite) (15). Kaplan Meier plot for progression-free survival was derived from the transcriptomic datasets with follow-up and clinical data from the GEO repository and were plotted with the interactive web server KMplot (16). HECTD4 gene expression levels in different breast cancer subtypes, the frequency of different HECTD4 mutations in breast cancer subtypes and HECTD4 mRNA expression in different tumor types were obtained from the pan-cancer data from The Cancer Genome Atlas (TCGA) (17). We used the pre-processed RNA sequencing gene expression file (EBPlusPlusAdjustPANCAN_IlluminaHiSeq_RNASeqV2.geneExp.tsv) from Pan-cancer atlas repository. The processed somatic mutation data was taken from the Pan-cancer atlas repository (mc3.v0.2.8.PUBLIC.maf.gz) which calculated by the MC3 collaboration aiming at a consensual and unified variant calling (18). We considered as somatically mutated if one of the following holds:

- The mutations were truncating ("Nonsense_Mutation", "Frame_Shift_Del", "Frame_Shift_Ins", "Translation_Start_Site", "Splice_Site")
- The nonsynonymous mutations ("Missense_Mutation", "In_Frame_Del", "In_Frame_Ins","Nonstop_Mutation") were "deleterious" in the SIFT field and "probably_damaging" in the PolyPhen.
- The nonsynonymous mutations were predicted to be "pathogenic" or "likely_pathogenic" by ClinVar (19).
- In any case an above mutation was labelled as "Benign" according to ClinVar we did not consider this mutation.

Germline mutations were processed by other groups (20) and obtained from GDC data portal (PCA_pathVar_integrated_filtered_adjusted.tsv), and the variants of unknown significance were excluded. Copy number ASCAT calls are obtained through (https://github.com/VanLoo-lab/ascat/) (21) and loss-of-heterozygosity is defined as minor allele being 0.

Correlation of HECTD4 and COX-2 across 21 Breast cancer cell lines

Publicly available cell line proteomics data was collected across 31 breast cell lines (22). A total of 21 breast cell lines were selected for which both the canonical HECTD4 and COX-2 proteins were quantified. A Pearson correlation test between HECTD4 and COX-2 across the cell lines was calculated using the cor.test function in R (23).

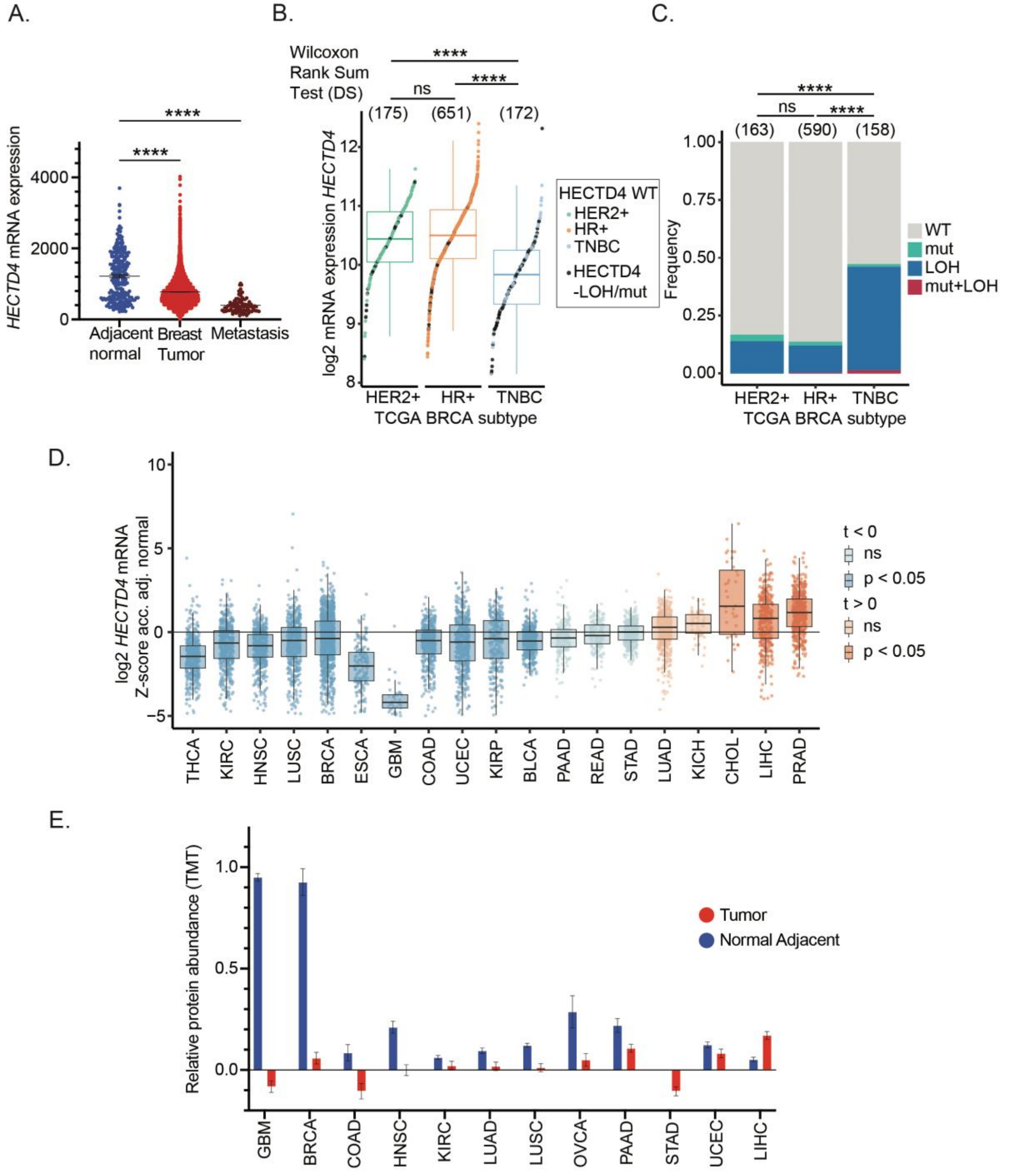

A.
HECTD4 mRNA expression
****
****
Adjacent normal
Breast Tumor
Metastasis
B.
Wilcoxon Rank Sum Test (DS)
****
ns
****
(175)
(651)
(172)
log2 mRNA expression HECTD4
HECTD4 WT
HER2+
HR+
TNBC
HECTD4 -LOH/mut
HER2+
HR+
TNBC
TCGA BRCA subtype
C.
****
ns
****
(163)
(590)
(158)
Frequency
WT
mut
LOH
mut+LOH
HER2+
HR+
TNBC
TCGA BRCA subtype
D.
log2 HECTD4 mRNA Z-score acc. adj. normal
t < 0
ns
p < 0.05
t > 0
ns
p < 0.05
THCA
KIRC
HNSC
LUSC
BRCA
ESCA
GBM
COAD
UCEC
KIRP
BLCA
PAAD
READ
STAD
LUAD
KICH
CHOL
LIHC
PRAD
E.
Relative protein abundance (TMT)
Tumor
Normal Adjacent
GBM
BRCA
COAD
HNSC
KIRC
LUAD
LUSC
OVCA
PAAD
STAD
UCEC
LIHC

Fig. S1. HECTD4 expression in clinical datasets (A) *HECTD4* gene expression is significantly decreased in primary and metastatic tumors compared with normal breast tissue (3691 normal breast tissues, 29,376 primary breast cancers and 453 metastatic breast cancers). Significance was calculated with one-way ANOVA test, with Dunnett's multiple comparison test. **(B)** *HECTD4* mRNA expression levels are compared across breast cancer subtypes with Wilcoxon Rank Sum test and p-values shown on top; loss of heterozygosity (LOH) or damaging/pathogenic point mutations are marked in black. **(C)** Frequency of *HECTD4* LOH and truncating, damaging or pathogenic single nucleotide variants (SNVs) and indels (SIFT or PolyPhen damaging, or ClinVar pathogenic or likely pathogenic) are shown in each breast cancer subtype. Fisher t-test was used to compare samples that are wild type (WT) or has mutation or LOH, p-values are shown on top of the bar plot. **(D)** *HECTD4* mRNA expression in each tumor type was Z-score normalized according to adjacent normal tissue, where negative value indicates a downregulation in tumor and a higher value indicates an upregulation; Z-score normalization is performed by subtracting the mean and dividing by the standard deviation in adjacent normal samples (tumor types with >3 adjacent normal samples are shown). T-test was performed, and p-values indicated by the color of boxes and markers were adjusted with the Benjamini-Hochberg procedure. Tumor types are sorted according to p-value, and the sign of the t-value with those with higher significance are at the two extremities. THCA: Thyroid cancer, KIRC: Kidney renal clear cell carcinoma, HNSC: Head and neck squamous cell carcinoma, LUSC: Lung squamous cell carcinoma, BRCA: Breast cancer, ESCA: Esophageal cancer, GBM: Glioblastoma, COAD: Colon adenocarcinoma, UCEC: Uterine corpus endometrial carcinoma, KIRP: Kidney renal papillary cell carcinoma, BLCA: Bladder urothelial carcinoma, PAAD: Pancreatic adenocarcinoma, READ: Rectum adenocarcinoma, STAD: Stomach adenocarcinoma, LUAD: Lung adenocarcinoma, KICH: Chromophobe renal cell carcinoma, CHOL: Cholangiocarcinoma, LIHC: Liver hepatocellular carcinoma, PRAD: Prostate adenocarcinoma **(E)** HECTD4 protein levels across different normal tissues and tumor types. Error bars represent mean +/- SD. GBM: Glioblastoma, BRCA: Breast cancer, COAD: Colon adenocarcinoma, HNSC: Head and neck squamous cell carcinoma, KIRC: Kidney renal clear cell carcinoma, LUAD: Lung adenocarcinoma, LUSC: Lung squamous cell carcinoma, OVCA: Ovarian carcinoma, PAAD: Pancreatic adenocarcinoma, STAD: Stomach adenocarcinoma, UCEC: Uterine corpus endometrial carcinoma, LIHC: Liver hepatocellular carcinoma. (* $p<0.05$, ** $p<0.01$, *** $p<0.001$, **** $p<0.0001$)

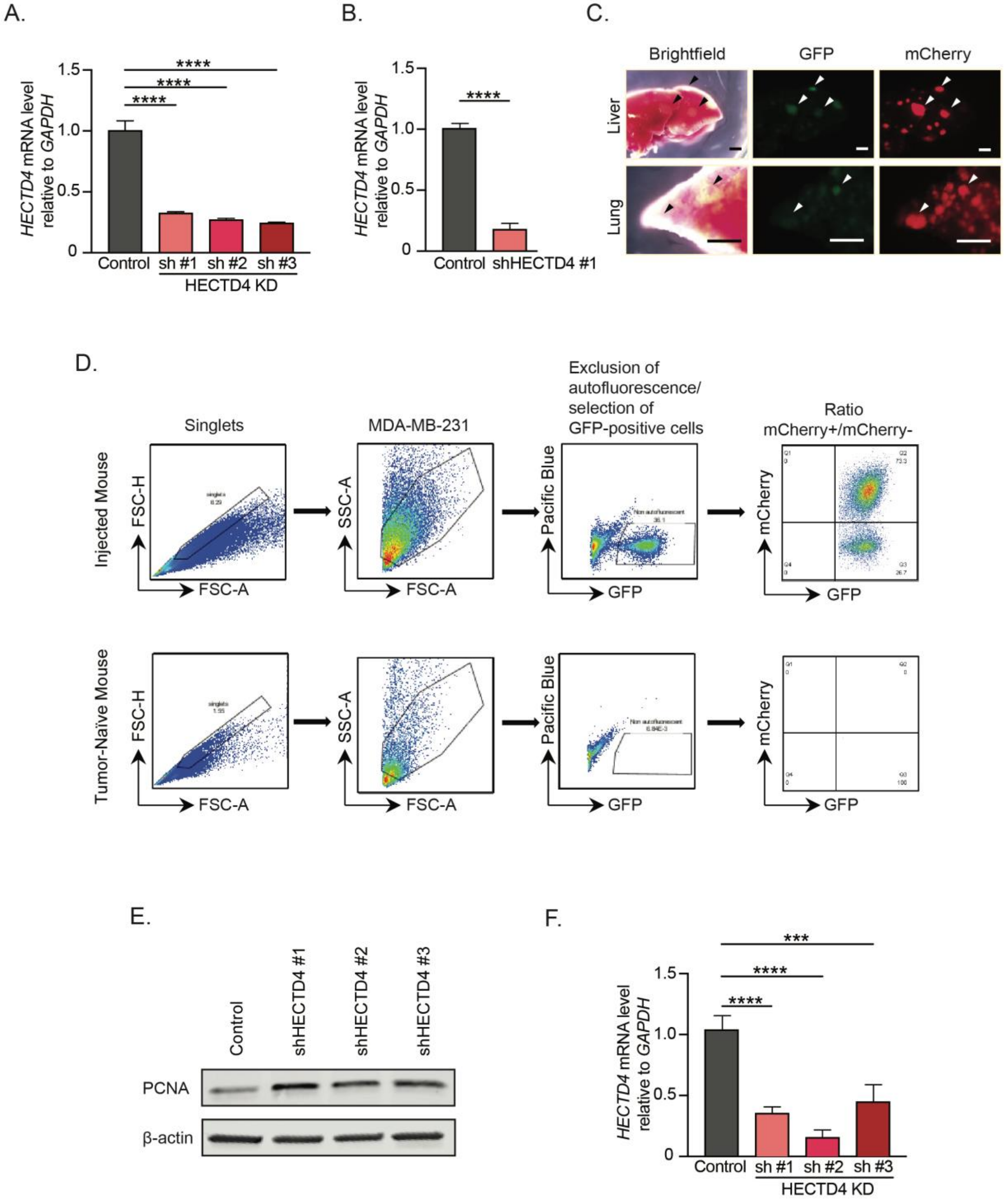
A.
HECTD4 mRNA level relative to GAPDH
1.5
1.0
0.5
0
****
****
****
Control
sh #1
sh #2
sh #3
HECTD4 KD
B.
HECTD4 mRNA level relative to GAPDH
1.5
1.0
0.5
0
****
Control
shHECTD4 #1
C.
Brightfield
GFP
mCherry
Liver
Lung
D.
Singlets
MDA-MB-231
Exclusion of autofluorescence/ selection of GFP-positive cells
Ratio mCherry+/mCherry-
Injected Mouse
Tumor-Naive Mouse
FSC-H
FSC-A
SSC-A
Pacific Blue
GFP
mCherry
E.
Control
shHECTD4 #1
shHECTD4 #2
shHECTD4 #3
PCNA
β-actin
F.
HECTD4 mRNA level relative to GAPDH
1.5
1.0
0.5
0
***
****
****
Control
sh #1
sh #2
sh #3
HECTD4 KD

Fig. S2. HECTD4 depletion increases tumor cell proliferation in vivo and under anchorage-independent conditions (A) shRNA-mediated *HECTD4* knockdown in MDA-MB-231 leads to reduced *HECTD4* mRNA levels compared to control cells transduced with scramble shRNA. Cells infected with shHECTD4 #1 were used for the experimental result shown **Fig. 2A.** Cells in which *HECTD4* was knocked down with all three viruses were used for experiments shown in **Fig. 2C.** Error bars represent mean +/- SD. Significance was calculated using one-way ANOVA test, with Dunnett's multiple comparison test. **(B)** shRNA-mediated *HECTD4* knockdown in MDA-MB-231 leads to reduced *HECTD4* mRNA levels compared to control cells transduced with scramble shRNA. These cells were used for the in vivo mixing experiment in **Fig. 2B.** Error bars represent mean +/- SD. Significance was calculated using unpaired student t-test. **(C)** Representative images of the liver and lungs from the experiment shown in **Fig. 2B** with macro-metastasis, in brightfield and the specific emission filter to expose GFP-fluorescent cells and mCherry-fluorescent cells. Tumor cells are indicated by arrow heads. Scale bar: 2 mm. **(D)** Flow cytometry gating procedure for the detection of GFP+/mCherry+ tumor cells in the tumor cell mixtures in the experiment indicated in **Fig. 2B**. The gating strategy is specific, with no tumor cells detected in a naïve mouse (lower line). **(E)** *HECTD4*-KD cells compared with control cells have increased levels of PCNA protein in suspension. Cells were cultured in suspension for 3 days before the protein levels were analyzed by Western blot. **(F)** shRNA-mediated *HECTD4* knockdown in MDA-MB-231 leads to reduced *HECTD4* mRNA levels compared to control cells transduced with scramble shRNA. These cells have been used in the soft agar experiments shown in **Fig. 2D.** Error bars represent mean +/- SD. Significance was calculated using one-way ANOVA test, with Dunnett's multiple comparison test. (* $p<0.05$, ** $p<0.01$, *** $p<0.001$, **** $p<0.0001$)

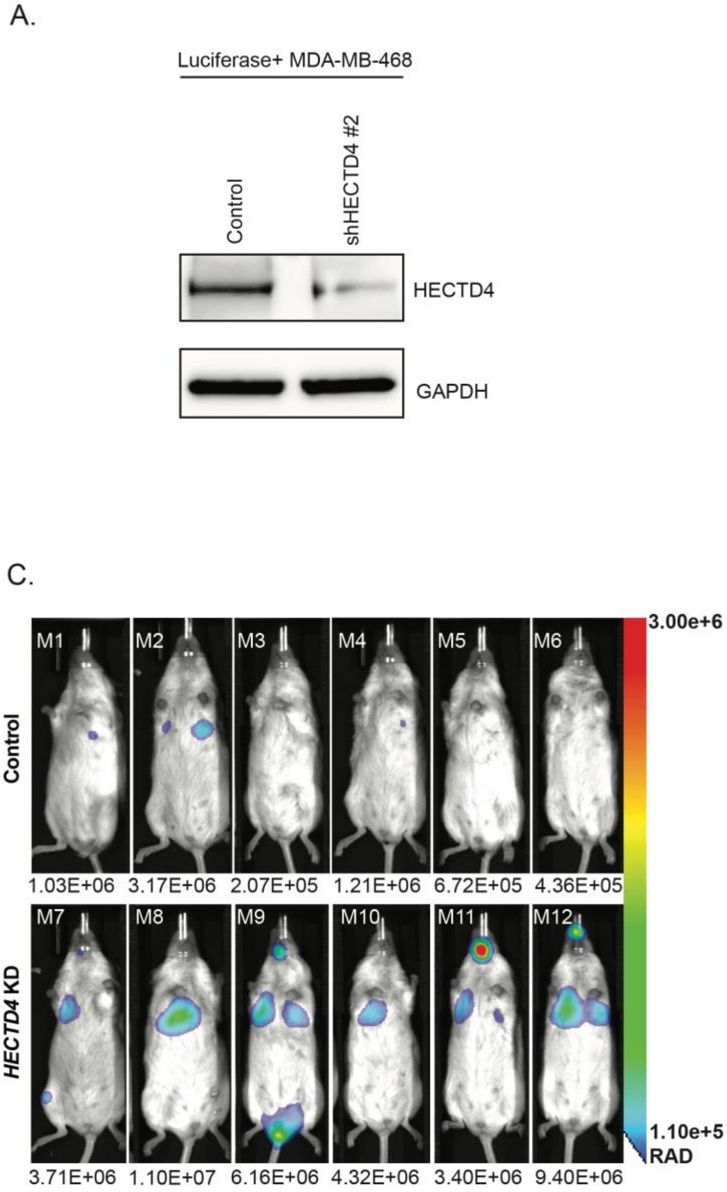
A.
Luciferase+ MDA-MB-468
Control
shHECTD4 #2
HECTD4
GAPDH
B.
Fold change relative to Day 0
15
10
5
0
****
Control
HECTD4 KD
C.
M1
M2
M3
M4
M5
M6
Control
1.03E+06
3.17E+06
2.07E+05
1.21E+06
6.72E+05
4.36E+05
M7
M8
M9
M10
M11
M12
HECTD4 KD
3.71E+06
1.10E+07
6.16E+06
4.32E+06
3.40E+06
9.40E+06
3.00e+6
1.10e+5
RAD
D.
Total flux (photons/sec)
1.5× 10^7
1.0× 10^7
5.0× 10^6
0.0
**
Control
HECTD4 KD

Fig. S3. MDA-MB-468 cells exhibit increased anchorage-independent growth and lung metastasis following knockdown of *HECTD4* (A) shRNA-mediated *HECTD4* (shHECTD4 #2) knockdown in Luciferase+ MDA-MB-468 cells results in reduced HECTD4 protein levels compared to cells transduced with scramble shRNA control. These cells were used in the experiments shown in **Fig. S3B-D. (B)** In vitro growth of *HECTD4*-depleted Luciferase+ MDA-MB-468 cells compared to scrambled control under anchorage-independent suspension conditions (ultra-low adherent plates). Cell viability and proliferation were measured by CellTiter Glo luminescence. Error bars represent mean +/- SD. Significance was calculated using unpaired student t-test on day 7. **(C)** Luciferase+ MDA-MB-468 cells harboring either the scramble shRNA control (M1-M6) or shHECTD4 #2 (M7-M12) were injected into the tail vein of immunosuppressed NSG mice. In vivo imaging system (IVIS) images from day 28 post-injection show bioluminescent signals mainly in the lung regions. Total flux values (photons/s) obtained from the lungs are reported under each image. The minimum to maximum radiance values is displayed on the color scale. **(D)** Bar graphs represent the total flux values (photons/sec) from the lung regions of individual mice (M1-M6, Control; M7-M12, *HECTD4*-KD) obtained by IVIS imaging on day 28 post-injection. Error bars represent mean +/- SD. Significance was calculated using unpaired student t-test. (* $p<0.05$, ** $p<0.01$, *** $p<0.001$, **** $p<0.0001$)

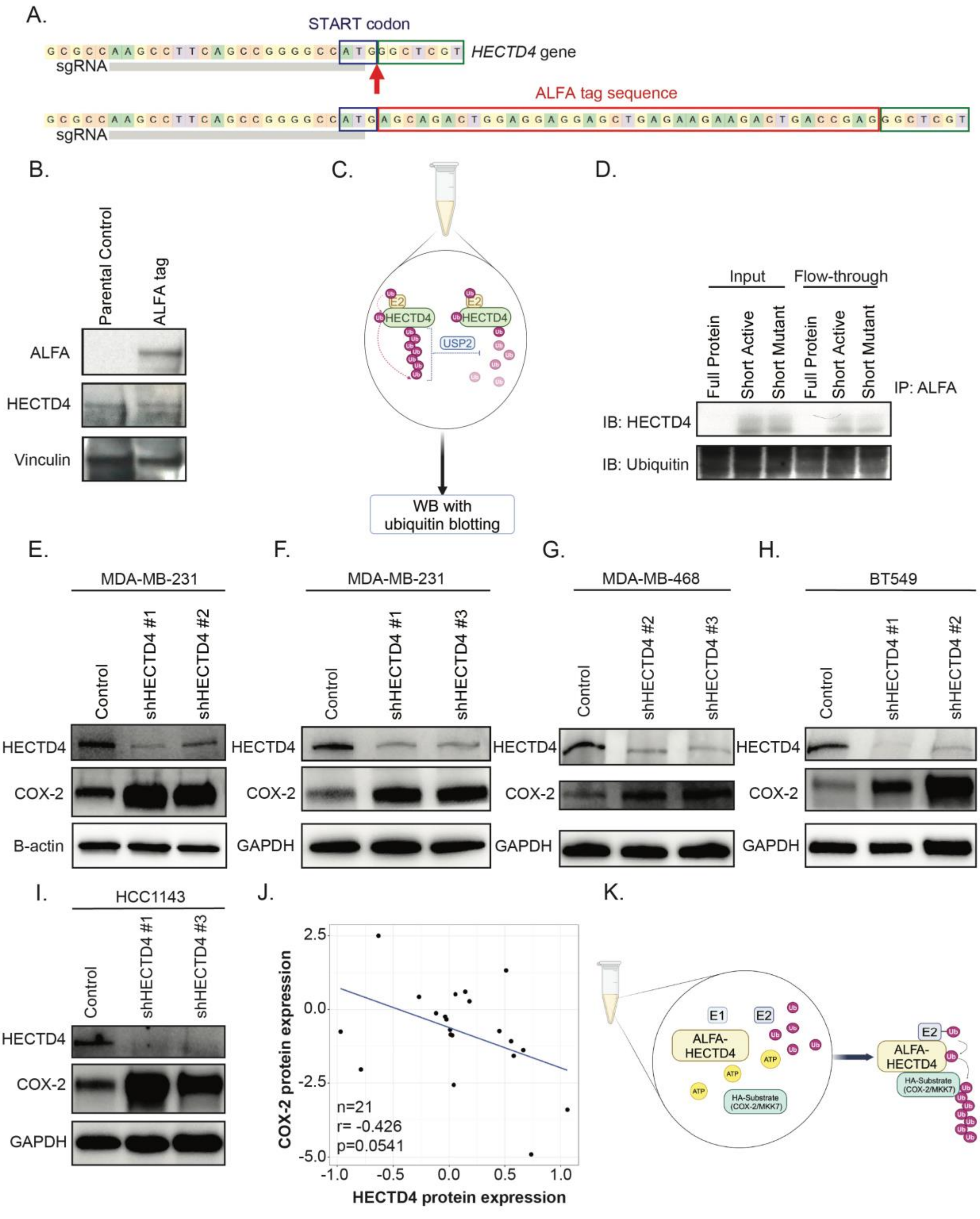

A.
START codon
G C G C C A A G C C T T C A G C C G G G G C C A T G G G C T C G T
HECTD4 gene
sgRNA
ALFA tag sequence
G C G C C A A G C C T T C A G C C G G G G C C A T G A G C A G A C T G G A G G A G G A G C T G A G A A G A A G A C T G A C C G A G G G C T C G T
sgRNA
B.
Parental Control
ALFA tag
ALFA
HECTD4
Vinculin
C.
E2
HECTD4
USP2
Ub
WB with ubiquitin blotting
D.
Input
Flow-through
Full Protein
Short Active
Short Mutant
Full Protein
Short Active
Short Mutant
IP: ALFA
IB: HECTD4
IB: Ubiquitin
E.
MDA-MB-231
Control
shHECTD4 #1
shHECTD4 #2
HECTD4
COX-2
B-actin
F.
MDA-MB-231
Control
shHECTD4 #1
shHECTD4 #3
HECTD4
COX-2
GAPDH
G.
MDA-MB-468
Control
shHECTD4 #2
shHECTD4 #3
HECTD4
COX-2
GAPDH
H.
BT549
Control
shHECTD4 #1
shHECTD4 #2
HECTD4
COX-2
GAPDH
I.
HCC1143
Control
shHECTD4 #1
shHECTD4 #3
HECTD4
COX-2
GAPDH
J.
COX-2 protein expression
2.5
0.0
-2.5
-5.0
-1.0
-0.5
0.0
0.5
1.0
HECTD4 protein expression
n=21
r= -0.426
p=0.0541
K.
E1
E2
Ub
ALFA-HECTD4
ATP
HA-Substrate (COX-2/MKK7)

Fig. S4. Ubiquitination and degradation of COX-2 by HECTD4 (A) The endogenous tagging of HECTD4 at the N-terminus with prime-editing CRISPR strategy (24). The plot shows the integration of the exogenous 27-bp ALFA sequence at the 3' of the START codon of the HECTD4 sequence. **(B)** Western blotting against the ALFA tag, detects HECTD4. Parental cells are shown as control. Western blotting for vinculin is shown as loading control. **(C)** Schematic representation of HECTD4-dependent ubiquitin chain formation in vitro. **(D)** The lysates from cells transfected with the short-active and short-mutant forms of alfa-HECTD4 were flowed through an ALFA-tag activated column. The input lysate, flow- through and the eluate were blotted and probed with antibodies against HECTD4 and ubiquitin. Untransfected parental cells are shown as control. The input lysate and flow-through are shown here, the elute is shown in **Fig. 3B (Lower)**. **(E)** Western blot showing increased COX-2 protein in *HECTD4*-KD (sh #1 and sh #2) MDA-MB-231 cells growing in suspension. **(F)** Western blot showing increased COX-2 protein in *HECTD4*-KD (sh #1 and sh #3) MDA-MB-231 cells growing in suspension. **(G)** Western blot showing increased COX-2 protein in *HECTD4*-KD (sh #2 and sh #3) MDA-MB-468 cells growing in suspension. **(H)** Western blot showing increased COX-2 protein in *HECTD4*-KD (sh #1 and sh #2) BT-549 cells growing in suspension. **(I)** Western blot showing increased COX-2 protein in *HECTD4*-KD (sh #1 and sh #3) HCC1143 cells growing in suspension. **(J)** Analysis of publicly available proteomics data from 21 human breast cancer cell lines (22) shows a negative correlation between HECTD4 and COX-2 protein levels ($N = 21$, $r = -0.426$, $p = 0.0541$). A Pearson correlation test between HECTD4 and COX-2 across the cell lines was calculated. **(K)** Schematic representation of HECTD4-dependent ubiquitin chain formation of the HA- tagged proteins in vitro. HA-tagged COX-2 and MKK7 were produced by in vitro transcription-translation in reticulocytes and incubated with active HECTD4, immunoprecipitated from freshly lysed cells using ALFA-tagged beads. E1, E2, ATP, HA-tagged COX-2 or MKK7, ALFA-tagged HECTD4 and ubiquitin were added into the reaction. The ubiquitination of the HA-tagged protein of interest was detected by western blotting.

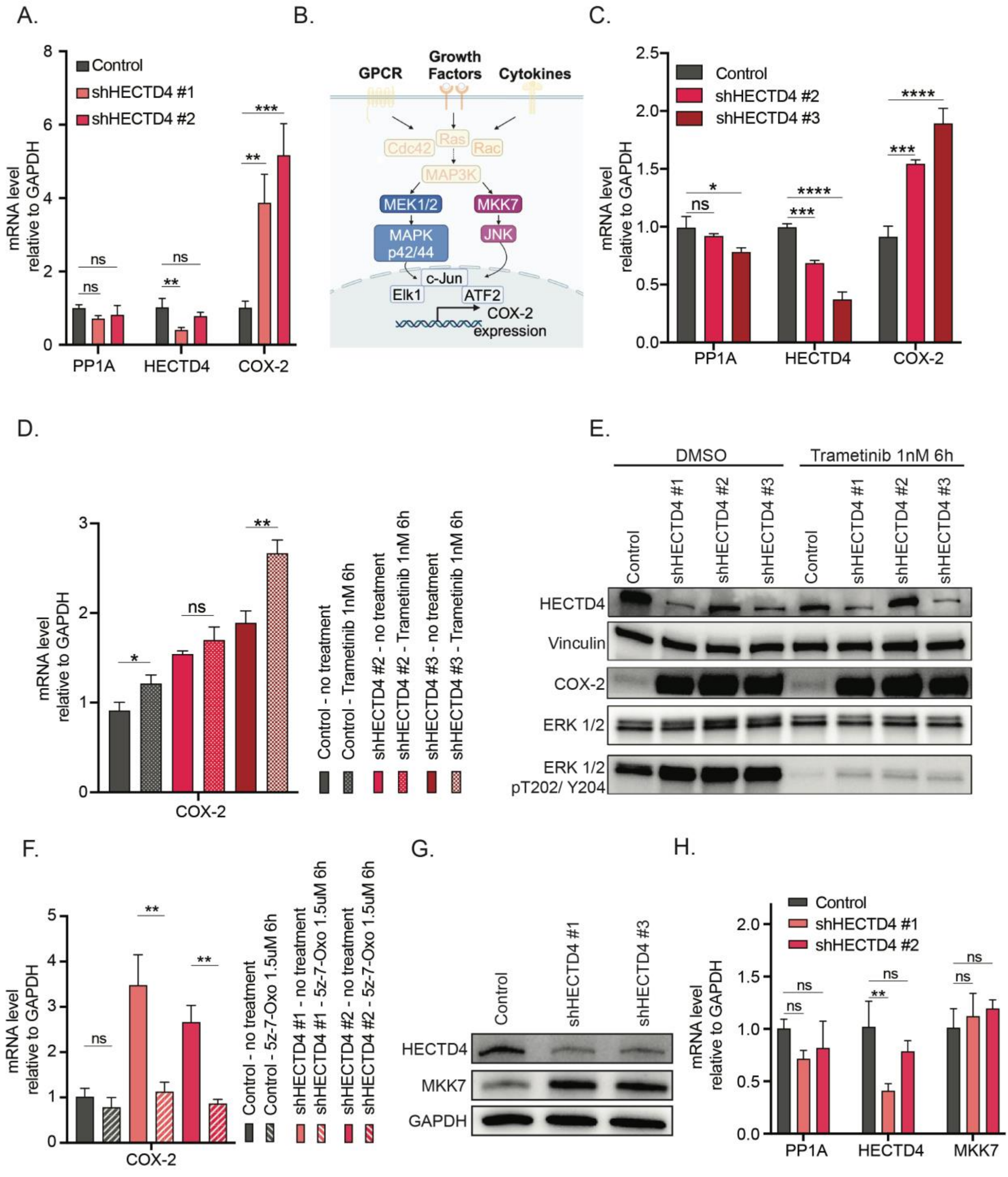
A.
B.
C.
D.
E.
F.
G.
H.
Control
shHECTD4 #1
shHECTD4 #2
shHECTD4 #3
mRNA level relative to GAPDH
PP1A
HECTD4
COX-2
MKK7
GPCR
Growth Factors
Cytokines
Cdc42
Ras
Rac
MAP3K
MEK1/2
MKK7
MAPK p42/44
JNK
c-Jun
Elk1
ATF2
COX-2 expression
Control - no treatment
Control - Trametinib 1nM 6h
shHECTD4 #2 - no treatment
shHECTD4 #2 - Trametinib 1nM 6h
shHECTD4 #3 - no treatment
shHECTD4 #3 - Trametinib 1nM 6h
DMSO
Trametinib 1nM 6h
Vinculin
ERK 1/2
ERK 1/2 pT202/ Y204
Control - 5z-7-Oxo 1.5uM 6h
shHECTD4 #1 - no treatment
shHECTD4 #1 - 5z-7-Oxo 1.5uM 6h
shHECTD4 #2 - 5z-7-Oxo 1.5uM 6h
GAPDH
ns
*
**
***
****

Fig. S5. HECTD4 targets the COX-2 regulator MKK7 (A) qRT-PCR of *PP1A*, *HECTD4* and *COX-2* mRNA in control and *HECTD4*-KD cells, showing that *COX-2* mRNA levels rise upon *HECTD4* depletion. Error bars represent mean +/- SD. Significance was calculated using one-way ANOVA test, with Dunnett's multiple comparison test. **(B)** Diagram showing the MAPK pathway regulating COX-2 transcription; selected players are highlighted. Several stimuli, including growth factors, proinflammatory cytokines, and environmental stress, activate the signaling cascade. MEK1 and MKK7 signal through parallel pathways, before converging to activate transcription factors (TF), such as Elk1, c-Jun and ATF2. These TF induce *COX-2* gene transcription. **(C)** qRT-PCR of *PP1A*, *HECTD4* and *COX-2* mRNA in control and *HECTD4*-KD cells, showing that *COX-2* mRNA levels rise upon *HECTD4* depletion. These cells have been used in the experiments shown in **Fig. S5D and Fig. 4B.** Error bars represent mean +/- SD. Significance was calculated using one-way ANOVA test, with Dunnett's multiple comparison test. **(D)** Trametinib was used to block MEK1 and investigate the effect of this inhibition on COX-2 activation. The qRT-PCR demonstrates that the drug fails to reverse *COX-2* mRNA induction in *HECTD4*-KD cells. Error bars represent mean +/- SD. Significance was calculated using unpaired student t-test. The baseline (no treatment) mRNA levels of *PP1A, HECTD4* and *COX-2* in cells used for this experiment are shown in **Fig. S5C.** The same cells were used in the experiments shown in **Fig. 4B. (E)** Western blot of the control and *HECTD4*-KD cells treated with trametinib. MEK1 is effectively inhibited, as demonstrated by the reduced activation of its direct target ERK 1/2 pT202/Y204. The increased COX-2 level in the *HECTD4*-KD is not affected by this inhibition, consistent with the qRT-PCR data shown in **Fig. S5D**. **(F)** qRT-PCR data demonstrates that MKK7 inhibition by the drug 5Z-7-oxozeanol (OXO) effectively reverses *COX-2* transactivation in *HECTD4*-KD cells. Error bars represent mean +/- SD. Significance was calculated using unpaired student t-test. **(G)** Western blot showing increased MKK7 protein in *HECTD4*-KD (sh #1 and sh #3) MDA-MB-231 cells growing in suspension. **(H)** *HECTD4* depletion does not impact *MKK7* mRNA levels, as shown by the qRT-PCR data from the same experiment in **Fig. S5A**. Error bars represent mean +/- SD. Significance was calculated using one-way ANOVA test, with Dunnett's multiple comparison test. (* $p<0.05$, ** $p<0.01$, *** $p<0.001$, **** $p<0.0001$)

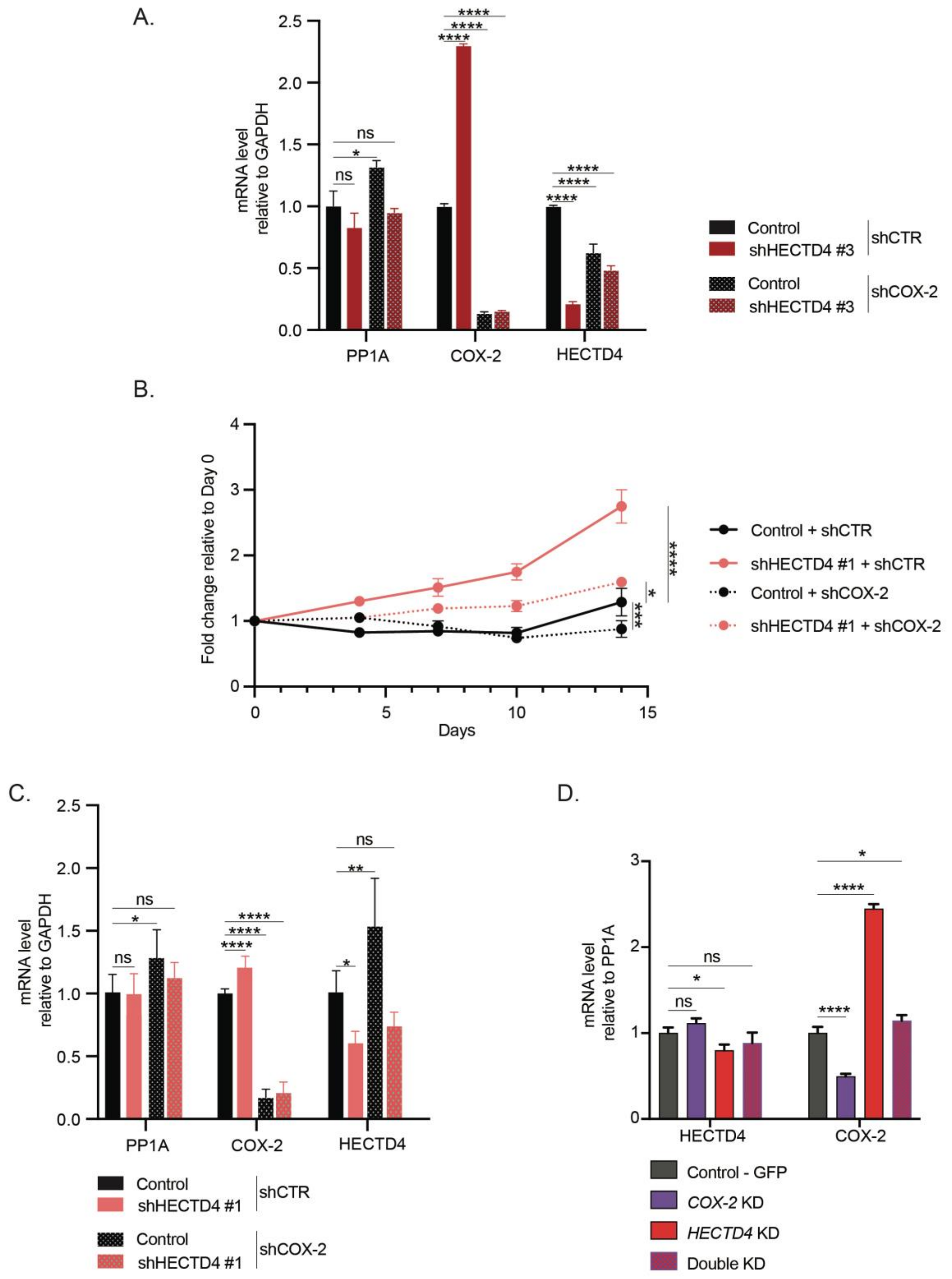
A.
mRNA level relative to GAPDH
2.5
2.0
1.5
1.0
0.5
0.0
ns
*
ns
****
****
****
****
****
****
PP1A
COX-2
HECTD4
Control
shHECTD4 #3
shCTR
Control
shHECTD4 #3
shCOX-2
B.
Fold change relative to Day 0
4
3
2
1
0
0
5
10
15
Days
****
*
***
Control + shCTR
shHECTD4 #1 + shCTR
Control + shCOX-2
shHECTD4 #1 + shCOX-2
C.
mRNA level relative to GAPDH
2.5
2.0
1.5
1.0
0.5
0.0
ns
*
ns
****
****
****
ns
**
*
PP1A
COX-2
HECTD4
Control
shHECTD4 #1
shCTR
Control
shHECTD4 #1
shCOX-2
D.
mRNA level relative to PP1A
3
2
1
0
ns
*
ns
*
****
****
HECTD4
COX-2
Control - GFP
COX-2 KD
HECTD4 KD
Double KD

Fig. S6. HECTD4 modulation of COX-2-dependent proliferation in vitro (A) qRT-PCR of *PP1A*, *HECTD4* and *COX-2* mRNA in control, *HECTD4*-KD, *COX-2*-KD and Double KD conditions. These cells were used in the experiments shown in **Fig. 4A.** Error bars represent mean +/- SD. Significance was calculated using one-way ANOVA test, with Dunnett's multiple comparison test. **(B)** Depletion of *HECTD4* compared to scrambled control increases cell proliferation under anchorage-independent, suspension conditions (ultra-low adherent culture dish). Depletion of *COX-2* in *HECTD4*-depleted cells reverts this phenotype. Cell viability and proliferation were measured by CellTiter Glo luminescence. Error bars represent mean +/- SD. Significance was calculated using with two-way ANOVA test, with Tukey's multiple comparison test (Day 14). Only cells harboring control and shHECTD4 #1 shRNA's are shown in this figure. Cells with control and shHECTD4 #3 shRNA's are shown in **Fig. 4A.** The mRNA levels of *PP1A, HECTD4* and *COX-2* in the cells used for this experiment are shown in **Fig. S6C. (C)** qRT-PCR of *PP1A*, *HECTD4* and *COX-2* mRNA in control, *HECTD4*-KD, *COX-2*-KD and Double KD conditions. These cells were used in the experiments shown in **Fig. S6B.** Error bars represent mean +/- SD. Significance was calculated using one-way ANOVA test, with Dunnett's multiple comparison test. **(D)** A mix of 2 shRNAs targeting *COX-2* efficiently reduced its mRNA level, both in the control and *HECTD4*-KD cells, reverting the HECTD4-mediated transactivation. These cells were used in the soft agar experiments shown in **Fig. 4C** and the in vivo mixing experiment shown in **Fig. 4D.** Error bars represent mean +/- SD. Significance was calculated using one-way ANOVA test, with Dunnett's multiple comparison test. (* $p<0.05$, ** $p<0.01$, *** $p<0.001$, **** $p<0.0001$)

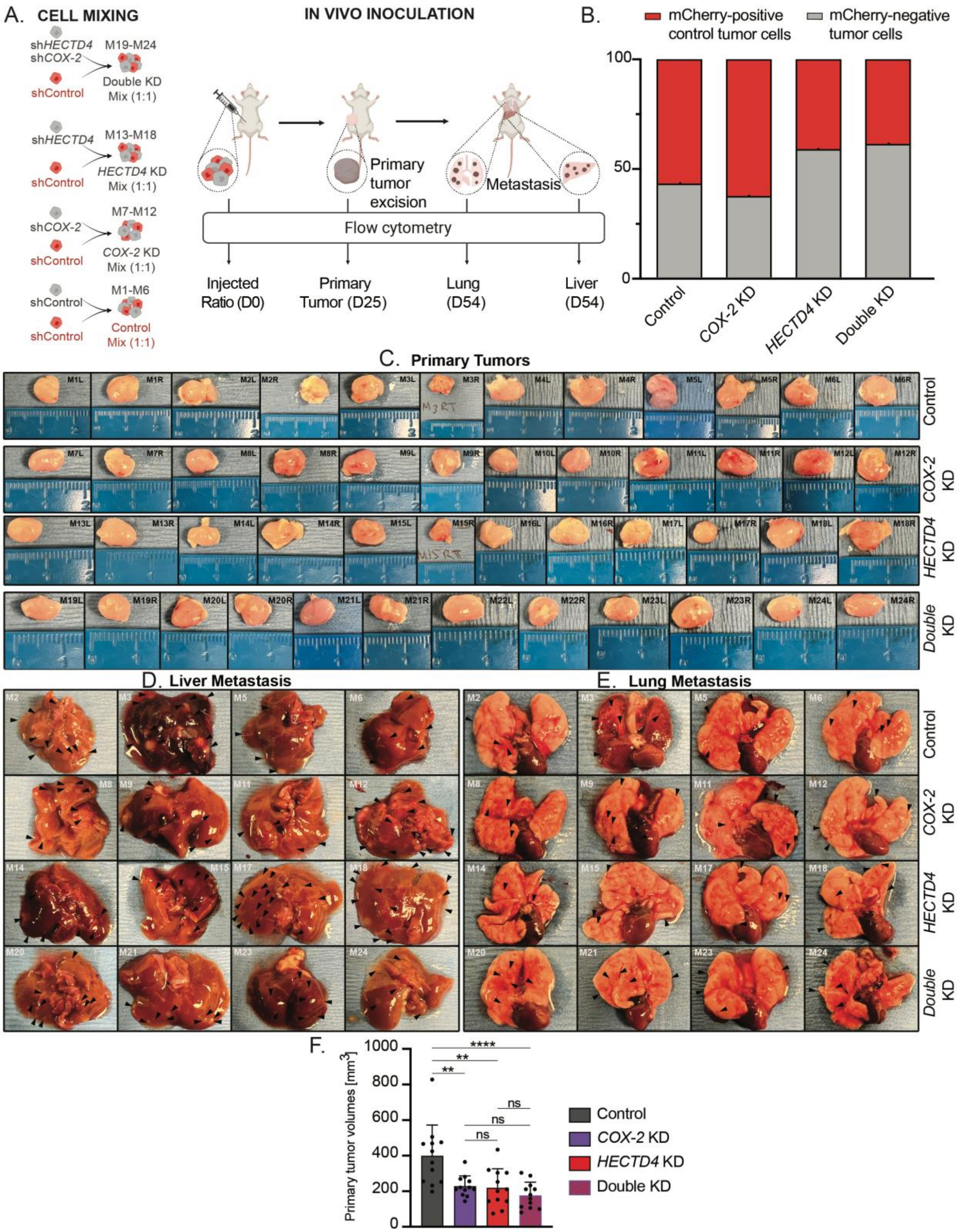
A. CELL MIXING
shHECTD4
shCOX-2
shControl
M19-M24
Double KD
Mix (1:1)
shHECTD4
shControl
M13-M18
HECTD4 KD
Mix (1:1)
shCOX-2
shControl
M7-M12
COX-2 KD
Mix (1:1)
shControl
shControl
M1-M6
Control
Mix (1:1)
IN VIVO INOCULATION
Primary tumor excision
Metastasis
Flow cytometry
Injected Ratio (D0)
Primary Tumor (D25)
Lung (D54)
Liver (D54)
B.
mCherry-positive control tumor cells
mCherry-negative tumor cells
100
50
0
Control
COX-2 KD
HECTD4 KD
Double KD
C. Primary Tumors
Control
COX-2 KD
HECTD4 KD
Double KD
D. Liver Metastasis
E. Lung Metastasis
F.
Primary tumor volumes [mm3]
1000
800
600
400
200
0
****
**
**
ns
ns
ns
Control
COX-2 KD
HECTD4 KD
Double KD

Fig. S7. HECTD4 modulation of COX-2-dependent tumorigenesis and metastasis (A) Schematic representation of the tumor cells mixing experiment conducted in vivo to assess the effect of COX-2 depletion in *HECTD4*-KD cells. GFP+/ mCherry+ control cells (GFP-mCherry-shControl; *HECTD4*-WT and *COX-2*-WT, shown in red) and four GFP+/mCherry- conditions i.GFP-shControl (*HECTD4*-WT and *COX-2*-WT, shown in gray), ii.GFP-*COX*-2-KD (*HECTD4*-WT and *COX-2*-KD, shown in gray), iii.GFP-*HECTD4*-KD (*HECTD4*-KD and *COX-2*-WT, shown in gray), iv.GFP-Double-KD (*HECTD4*-KD and *COX-2*-KD, shown in gray) were combined in a ratio of 1:1 to give rise to four mixtures, respectively the control group (M1-M6), *COX-2*-KD group (M7-M12), *HECTD4*-KD group (M13-M18) and Double KD group (M19-M24). The mixtures were separately injected into the mammary fat pads of NSG mice. The mixtures were also seeded in vitro and analyzed by flow cytometry to ensure that each of the populations were equally represented in the mixture inoculated into the mammary fat pad at day 0. The primary tumors were resected at day 25 to allow mouse survival and generation of metastases, and at day 54, mice were sacrificed and the colonized organs – lungs and livers – were harvested for analysis. The ratio of the different tagged cell populations was analyzed by flow cytometry. The ratios are shown in **Fig. 4D. (B)** Fraction of the GFP+ /mCherry- tumor cells in the tumor cell mixtures at day 0 just before injecting into the mammary fat pads of NSG mice. The bars represent the percentage of red (GFP-mCherry-shControl) and green (i.GFP-shControl, ii.GFP-*COX-2*-KD, iii.GFP-*HECTD4*-KD, iv.GFP-Double-KD, shown in gray) cells. **(C)** Representative images of the primary resected tumors from the experiment shown in **Fig. S7A.** Each row corresponds to a different experimental group. **(D)** Representative images of liver metastases at the time of termination from the experiment shown in **Fig. S7A**. Each row corresponds to a different experimental group. The black arrows indicate the metastatic nodules. **(E)** Representative images of lung metastases from the experiment shown in **Fig. S7A.** Each row corresponds to a different experimental group. The black arrows indicate the metastatic nodules. **(F)** The volumes of the primary tumors in the control, *COX-2*-KD, *HECTD4*-KD and Double KD groups upon resection at day 25. Error bars represent mean +/- SD. Significance was calculated using with one-way ANOVA test, with Tukey's multiple comparison test. (* $p<0.05$, ** $p<0.01$, *** $p<0.001$, **** $p<0.0001$)

Table S1. List of target sequences of the short hairpins

Gene	Manuscript name	Target sequence	Addgene #
HECTD4	sh #1	CGCTGCCTGTACCTTAGATTT	
HECTD4	sh #2	TGCGGAAGACACCCATATATA	
HECTD4	sh #3	GCGTCAGACACATTGACTATT	
COX-2	sh #1	GCTGAATTTAACACCCTCTAT	
COX-2	sh #2	CTATCACTTCAAACTGAAATT	
Control/Scramble	CTR	CCTAAGGTTAAGTCGCCCTCG	136035

Table S2. List of guideRNA for ALFA introduction at the N-terminus of HECTD4

Type	Sequence (including SpCas9 sgRNA scaffold)
pegRNA	5’- GAAGCCTTCAGCCGGGGCCATGTTTTAGAGCTAGAAATAGCAAGTTAAAATAAG GCTAGTCCGTTATCAACTTGAAAAAGTGGCACCGAGTCGGTGCGCCGCCGCGG CCGCCGACGAGCCCTCGGTCAGTCTTCTTCTCAGCTCCTCCTCCAGTCTGCTCA TGGCCCCGGCTGTTTTTTT -3’
ngRNA	5’- GCTCCAGTCTGCTCATGGCCCGTTTTAGAGCTAGAAATAGCAAGTTAAAATAAG GCTAGTCCGTTATCAACTTGAAAAAGTGGCACCGAGTCGGTGCTTTTTTT -3’

Table S3. List of primers used for qRT-PCR.

Gene	Forward primer	Reverse primer
GAPDH	5’-CCTCAACGACCACTTTGTCAAG-3’	5’-TGTTGCTGTAGCCAAATTCGTT- 3’
PP1A	5’-CATCTGCACTGCCAAGACTGAG-3’	5’-TCTTTCACTTTGCCAAACACCA- 3’
HECTD4	5’-AGGTACAGTGTGAACGGCTG-3’	5’-AGAACATGCAGGCTCGAACA- 3’
COX-2	5’-CTGGCGCTCAGCCATACAG-3’	5’-CGCACTTATACTGGTCAAATCCC-3’
MKK7	5’-GAAGGAGAAGCACGGTGTCA-3’	5’-CTCCACCTGGGAGGGAGAC- 3’

Table S4. List of antibodies used for Western Blot

Protein/Target	Company	Cat#	Concentration
HECTD4	ThermoFisher	PA5-61010	1:1000
Ubiquitin	Cell Signaling	14049S	1:1000
Vinculin	Sigma	MAB3574	1:1000
ALFA tag	Nanotag Bio.	N1505-HRP	1:3000
β-actin	Cell Signaling	3700S	1:1000
COX-2	Cell Signaling	12282S	1:1000
HA tag	Sant Cruz Bio.	SC-7392	1:200
MKK7	Cell Signaling	4172S	1:1000
MAPK total (Erk1)	Cell Signaling	4695T	1:1000
Phospho-MAPK (T202/Y204)	Cell Signaling	4370T	1:1000
PCNA	Cell Signaling	13110S	1:1000

SI References

1. N. V. Jordan *et al.*, HER2 expression identifies dynamic functional states within circulating breast cancer cells. *Nature* **537**, 102-106 (2016).
2. M. Yu *et al.*, Cancer therapy. Ex vivo culture of circulating breast tumor cells for individualized testing of drug susceptibility. *Science* **345**, 216-220 (2014).
3. R. Y. Ebright *et al.*, Deregulation of ribosomal protein expression and translation promotes breast cancer metastasis. *Science* **367**, 1468-1473 (2020).
4. K. R. Sanson *et al.*, Optimized libraries for CRISPR-Cas9 genetic screens with multiple modalities. *Nat Commun* **9**, 5416 (2018).
5. A. Edwards, W. Haas, Multiplexed Quantitative Proteomics for High-Throughput Comprehensive Proteome Comparisons of Human Cell Lines. *Methods Mol Biol* **1394**, 1-13 (2016).
6. M. Kathiresan *et al.*, Protein interactome homeostasis through an N-recognin E3 ligase is a vulnerability in aneuploid cancer. *BioXRiv* 10.1101/2023.05.04.539299 (2023).
7. L. Ting, R. Rad, S. P. Gygi, W. Haas, MS3 eliminates ratio distortion in isobaric multiplexed quantitative proteomics. *Nat Methods* **8**, 937-940 (2011).
8. G. C. McAlister *et al.*, MultiNotch MS3 enables accurate, sensitive, and multiplexed detection of differential expression across cancer cell line proteomes. *Anal Chem* **86**, 7150-7158 (2014).
9. E. L. Huttlin *et al.*, A tissue-specific atlas of mouse protein phosphorylation and expression. *Cell* **143**, 1174-1189 (2010).
10. J. E. Elias, S. P. Gygi, Target-decoy search strategy for increased confidence in large-scale protein identifications by mass spectrometry. *Nat Methods* **4**, 207-214 (2007).
11. M. E. Ritchie *et al.*, limma powers differential expression analyses for RNA-sequencing and microarray studies. *Nucleic Acids Res* **43**, e47 (2015).
12. Y. Benjamini, Y. Hochberg, Controlling the False Discovery Rate: A Practical and Powerful Approach to Multiple Testing. *Journal of the Royal Statistical Society: Series B (Methodological)* **57**, 289-300 (1995).
13. N. Saitou, M. Nei, The neighbor-joining method: a new method for reconstructing phylogenetic trees. *Molecular Biology and Evolution* **4**, 406-425 (1987).
14. A. Bartha, B. Gyorffy, TNMplot.com: A Web Tool for the Comparison of Gene Expression in Normal, Tumor and Metastatic Tissues. *Int J Mol Sci* **22** (2021).
15. D. Wang *et al.* (2022) cProSite: A web based interactive platform for on-line proteomics and phosphoproteomics data analysis. in *AACR*, ed P. o. t. t. A. M. o. t. A. A. f. C. Research (New Orlans, Louisiana (LA)).
16. B. Gyorffy, Survival analysis across the entire transcriptome identifies biomarkers with the highest prognostic power in breast cancer. *Comput Struct Biotechnol J* **19**, 4101-4109 (2021).
17. N. Cancer Genome Atlas Research *et al.*, The Cancer Genome Atlas Pan-Cancer analysis project. *Nat Genet* **45**, 1113-1120 (2013).
18. K. Ellrott *et al.*, Scalable Open Science Approach for Mutation Calling of Tumor Exomes Using Multiple Genomic Pipelines. *Cell Syst* **6**, 271-281 e277 (2018).

19. M. J. Landrum *et al.*, ClinVar: improving access to variant interpretations and supporting evidence. *Nucleic Acids Res* **46**, D1062-D1067 (2018).
20. K. L. Huang *et al.*, Pathogenic Germline Variants in 10,389 Adult Cancers. *Cell* **173**, 355-370 e314 (2018).
21. E. M. Ross, K. Haase, P. Van Loo, F. Markowetz, Allele-specific multi-sample copy number segmentation in ASCAT. *Bioinformatics* **37**, 1909-1911 (2021).
22. D. P. Nusinow *et al.*, Quantitative Proteomics of the Cancer Cell Line Encyclopedia. *Cell* **180**, 387-402 e316 (2020).
23. R Core Team (2021) R: A language and environment for statistical computing. (R Foundation for Statistical Computing, Vienna, Austria).
24. A. V. Anzalone *et al.*, Search-and-replace genome editing without double-strand breaks or donor DNA. *Nature* **576**, 149-157 (2019).
